# Construction of AMPS/ODMA-Modified Silica Composite Gel System and Its Applicability Evaluation in High-Temperature and High-Salinity Reservoirs

**DOI:** 10.3390/gels12090849

**Published:** 2026-09-18

**Authors:** Yang Zhao, Penghui Dai, Xingwang Wang, Yuhao Li, Jingchun Wu, Zhihua Guo, Chengke Ren

**Affiliations:** 1 Key Laboratory for EOR Technology (Ministry of Education), College of Petroleum Engineering, Northeast Petroleum University, Daqing 163318, China; dqzy0683@163.com (Y.Z.); d15822787509@163.com (P.D.); wujingchun9200@163.com (J.W.) 13290255005@163.com (C.R.); 2 Petroleum Exploration and Production Research Institute, China Petroleum & Chemical Corporation, Beijing 100083, China; xingwang.syky@sinopec.com; 3 Daqing International Exploration and Development Company, Daqing 163318, China; lyh_0218@163.com; 4 School of Earth Sciences, Northeast Petroleum University, Daqing 163318, China

**Keywords:** nano-SiO_2_, bifunctional modification, composite gel system, hydrophobic association, high temperature and high salt reservoir, enhanced oil recovery

## Abstract

In high-temperature and high-salinity reservoirs, HPAM is prone to thermal degradation, salt-induced chain shrinkage and shear instability, which weakens its mobility-control ability. In view of the above problems, this paper prepared bifunctional modified nano-SiO_2_ (KH570@SiO_2_-AMPS/ODMA, DF-SiO_2_), and constructed a composite gel system with HPAM. Its performance was evaluated by structural and rheological characterization, aging and shear recovery, and heterogeneous core displacement. At 90 °C and about 20,000 mg/L salinity, the viscosity retention rate exceeded 70% after 90 days of aging, and recovered to 94.6% of the initial value after 72 h of standing following shearing. Core displacement shows that the system can improve the flow distribution of heterogeneous reservoirs, and the recovery of high and low permeability layers is 6.95 and 4.41 percentage points higher than that of HPAM, respectively. DF-SiO_2_ constructs a dynamic network through interfacial interaction and hydrophobic association to improve the stability and structural recovery ability of the system, which provides a new idea for the application of high-temperature and high-salinity heterogeneous reservoirs.

## 1. Introduction

Polymer flooding can increase the viscosity of injected water, improve the mobility ratio and expand the swept volume, so it has become one of the more mature chemical flooding technologies in improving oil recovery in high water cut oilfields [1,2,3]. However, with the expansion of polymer flooding to high-temperature, high-salt and strong heterogeneous reservoirs, the structural stability of traditional partially hydrolyzed polyacrylamide (HPAM) systems in long-term use has gradually emerged [2,4,5,6]. At high temperature, the movement of polymer chain segments is intensified and accompanied by thermal degradation. The ion shielding at high salinity weakens the electrostatic repulsion between chains, so that the polymer chain changes from relative extension to contraction, which in turn reduces the viscoelasticity and mobility-control ability of the system [7,8]. For the network-type displacement system based on chain entanglement and intermolecular physical interaction, temperature, salinity and shear disturbance not only affect the macroscopic viscosity, but also directly affect the integrity and stability of its internal network structure. Therefore, the construction of a polymer gel system with stable spatial network and long-term structural integrity under complex reservoir conditions is the core issue in the design of chemical flooding materials for high-temperature and high-salinity reservoirs.

Nanoparticles have a large specific surface area, abundant surface active sites, and strong interfacial regulation ability, which provide an effective way to construct polymer composite gel systems [9,10,11]. SiO_2_ is widely used in the structure control of polymers and gels due to its wide source, high thermal stability and easy surface functionalization [12,13,14]. Previous studies have shown that SiO_2_ can participate in the interaction between polymer chains through surface adsorption, interface interaction and physical connection, promote the formation of spatial network structure and improve rheological stability [15,16,17]. Further surface functionalization of SiO_2_ can regulate the interfacial interaction between SiO_2_ and polymer chains, so that nanoparticles are no longer just inorganic reinforcing components, but become functional nodes involved in the construction of gel networks [18,19,20]. Previous studies have further shown that different surface modification methods can endow SiO_2_ with different interfacial interaction characteristics. Corredor et al. [21] used octyltriethoxysilane, oleic acid and stearic acid to modify the surface of SiO_2_, and investigated the interaction between different modified nanoparticles and HPAM and its effect on the rheology and oil displacement performance of the system. Haruna et al. [22] used APTES to modify the surface of SiO_2_, and enhanced the interaction between nanoparticles and polyacrylamide by introducing positively charged active groups, which improved the stability of the system under high-temperature and high-salt conditions. Liu et al. [17] introduced nano-SiO_2_ into the hydrophobically associating polyacrylamide system, indicating that nano-SiO_2_ can participate in the construction of polymer networks and improve the viscoelasticity, temperature resistance and shear resistance of the system.

These studies show that the surface functionalization of SiO_2_ can effectively improve the macroscopic properties of HPAM-based systems by changing the surface properties of particles and the particle–polymer interface. On this basis, the introduction of functional groups with different action characteristics can further regulate the interfacial interactions between nanoparticles and polymers, and optimize the performance of composite gel systems.

In the high-temperature and high-salt environment, the stability of the polymer network not only depends on the initial structural strength, but also is closely related to the molecular chain conformation and the network connection. High salinity will compress the electrostatic repulsion between polymer segments, shrink the molecular chain and weaken the effective network structure. At the same time, continuous thermal effects and shear disturbances may also cause damage to the physical connection points. Therefore, for gel systems based on physical interactions, both the stability of network connections and their recovery characteristics after exposure to external environments need to be considered. Hydrophobic groups can form reversible physical crosslinks via interchain hydrophobic association and establish associations between molecular chains [17,18,19]; structures containing sulfonic acid groups possess strong hydration capacity and ionic tolerance, which helps improve the stability of the system under high-salinity conditions [18,23,24]. It can be concluded that the dynamic connection provided by the hydrophobic association and the environmental stability provided by the salt-tolerant structure have a potential complementary effect. Based on this understanding, different functions are introduced into the same nano-interface, and the multi-level composite network is further constructed by using the interface connection of nanoparticles, which provides a possible way to improve the structural stability and dynamic response ability of the gel system under complex reservoir conditions.

To this end, this study constructed a HPAM-based composite gel system for a high-temperature and high-salt reservoir environment. KH570@SiO_2_-ODMA/AMPS-modified nano-SiO_2_ (hereinafter referred to as DF-SiO_2_) was prepared by introducing salt-resistant monomer 2-acrylamide-2-methylpropanesulfonic acid (AMPS) and hydrophobically associating monomer octadecyl methacrylate (ODMA) on the surface of particles. KH570 was used to introduce further-reactive organic linkage sites on the surface of SiO_2_, so that AMPS and ODMA could be introduced into the surface of nanoparticles. Among them, AMPS helps to improve the hydration stability under high-salinity conditions, and ODMA forms a reversible physical connection through hydrophobic association. The functionalized SiO_2_ further interacts with HPAM and participates in the formation of composite gel network. Previous studies have shown that different surface functionalization methods can regulate the interfacial interaction between SiO_2_ and HPAM. On this basis, this study further combined salt resistance, hydrophobic association and particle–polymer interface into the same composite gel system, and investigated its effects on the viscoelasticity, temperature and salt resistance, post-shear structure recovery ability, long-term stability and flow control behavior of porous media. Further analysis of the internal relationship between network structure stability and heterogeneous reservoir profile control and flooding provides ideas for the functional design of composite gel system and enhanced oil recovery in high temperature and high salinity reservoirs.

## 2. Results and Discussion

### 2.1. Structure and Interface Characteristics of KH570@SiO_2_-AMPS/ODMA

#### 2.1.1. FT-IR

**Figure 1:**
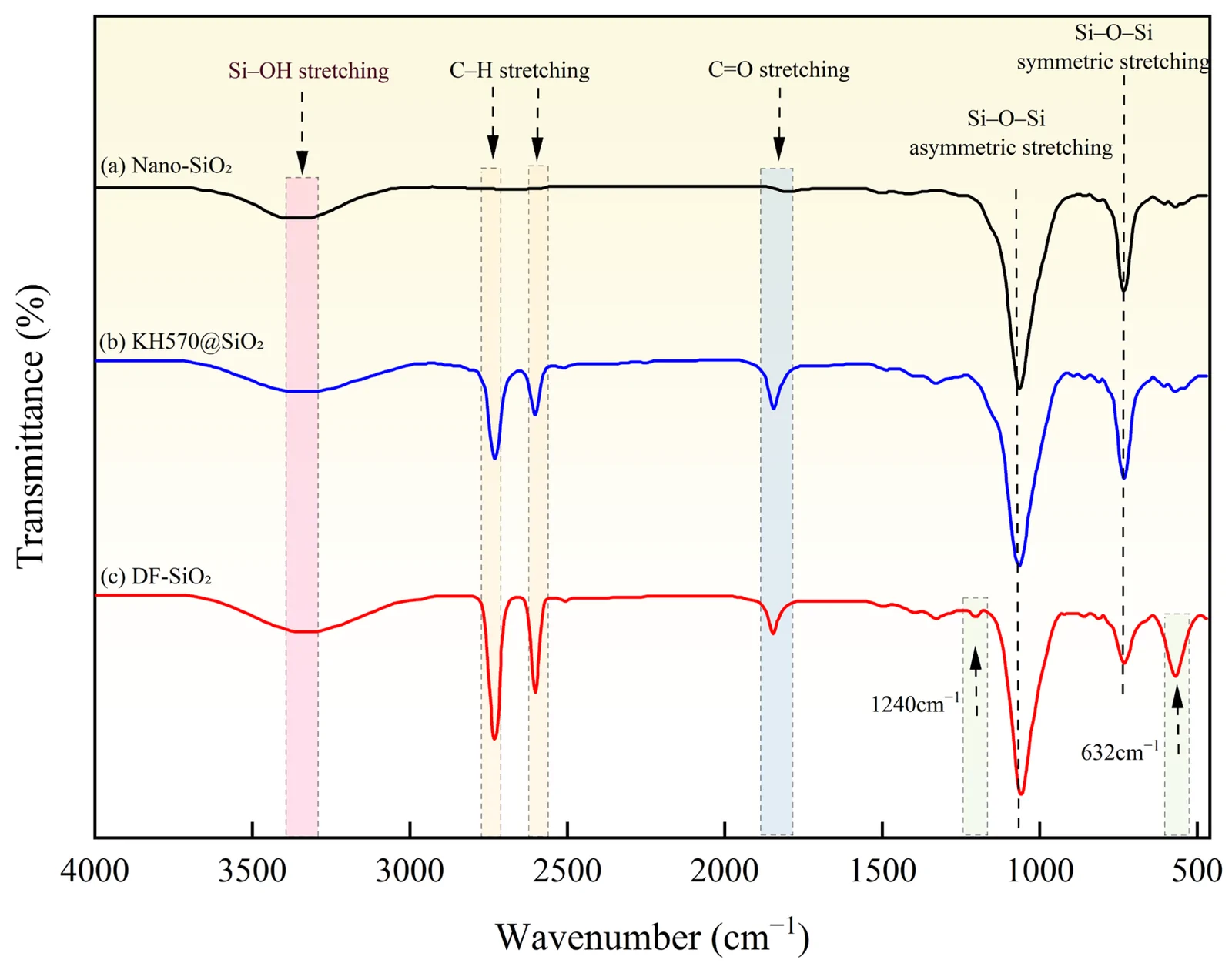
FT-IR spectra of different nano-SiO_2_ samples.

Figure 1 shows the FT-IR spectra of Nano-SiO_2_, KH570@SiO_2_ and KH570@SiO_2_AMPS/ODMA (DF-SiO_2_).

The characteristic absorption of Nano-SiO_2_ near 1100 and 805 cm^−1^ mainly corresponds to the Si-O-Si skeleton vibration, and the broad peak near 3426 cm^−1^ is related to the surface hydroxyl vibration [12,18]. After KH570 modification, the C-H stretching vibration absorption appeared near 2925 and 2855 cm^−1^, and the C=O characteristic absorption appeared near 1720 cm^−1^, and the surface hydroxyl absorption was weakened, indicating that KH570 formed a stable connection with the SiO_2_ surface through hydrolysis condensation [19,25]. After further grafting of AMPS and ODMA, a new sulfonic acid group-related characteristic absorption of DF-SiO_2_ appears near 1240 cm^−1^, and the C-H absorption intensity is further enhanced, corresponding to the introduction of long-chain alkyl structure. In addition, compared with KH570@SiO_2_, the C=C characteristic absorption of DF-SiO_2_ near 1630 cm^−1^ is significantly weakened, indicating that the polymerizable double bond retained by KH570 is involved in the subsequent free radical grafting reaction. The absorption peak near 632 cm^−1^ still exists before and after modification, indicating that the Si-O-Si skeleton is maintained during the functionalization process [14,18].

The continuous changes in the above characteristic peaks indicate that KH570 bridging and AMPS/ODMA functionalized grafting change the chemical composition of the surface of SiO_2_ particles, and an organic functional layer containing sulfonic acid groups and long-chain alkyl groups is formed outside the inorganic SiO_2_ skeleton.

Therefore, DF-SiO_2_ not only has a stable inorganic framework, but also has a surface functional structure that can interact with HPAM and hydrophobic association, which provides a chemical structure basis for the subsequent construction of a dynamic composite gel system.

#### 2.1.2. TGA

**Figure 2:**
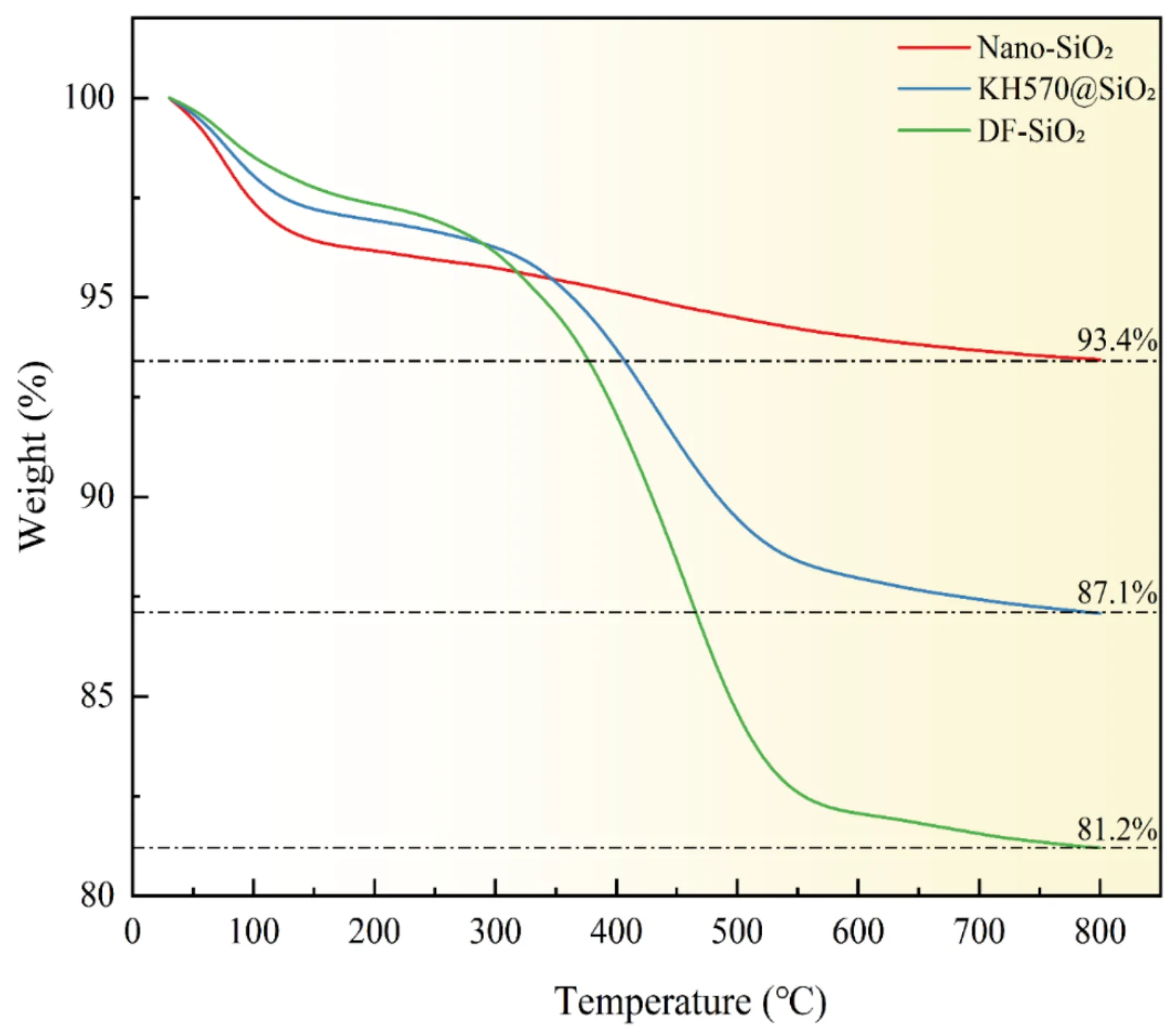
TGA curves of different nano-SiO_2_ samples.

Figure 2 shows the TGA curves of Nano-SiO_2_, KH570@SiO_2_ and DF-SiO_2_ in N_2_ atmosphere. The three samples all showed the characteristics of gradual weight loss with the increase in temperature, but there were significant differences in the weight loss of samples with different degrees of modification. Nano-SiO_2_ shows high thermal stability in the whole test temperature range, and the residual mass is 93.4% after 800 °C. A small amount of weight loss is mainly related to the process of surface adsorbed water removal and silanol condensation [12,14].

After KH570 modification, the residual mass of the sample at 800 °C decreased to 87.1%, and there was a significant weight loss stage in the range of 250–500 °C, corresponding to the thermal decomposition of the organic silane chain segment on the surface of the particles [19,20]. After further introduction of AMPS and ODMA, the residual mass of DF-SiO_2_ at 800 °C was further reduced to 81.2%, and the total weight loss reached 18.8%, which was about 5.9% higher than that of KH570@SiO_2_. The new weight loss is mainly related to the thermal cracking of the surface grafted functional segments, indicating that the AMPS/ODMA functional layer further increases the content of organic components on the surface of the particles.

With the development of modification from KH570 bridging to further functionalization of AMPS/ODMA, the weight loss behavior of the samples showed a continuous change, indicating that the organic functional layer on the nano-SiO_2_ increased gradually. Combined with the FT-IR results, it can be further confirmed that DF-SiO_2_ has formed a composite surface structure composed of SiO_2_ inorganic skeleton, KH570 interface layer and AMPS/ODMA functional segment. This structure provides a material basis for the establishment of interfacial association between nanoparticles and HPAM, and is conducive to the construction and stability of subsequent dynamic network structures.

#### 2.1.3. XPS

**Figure 3:**
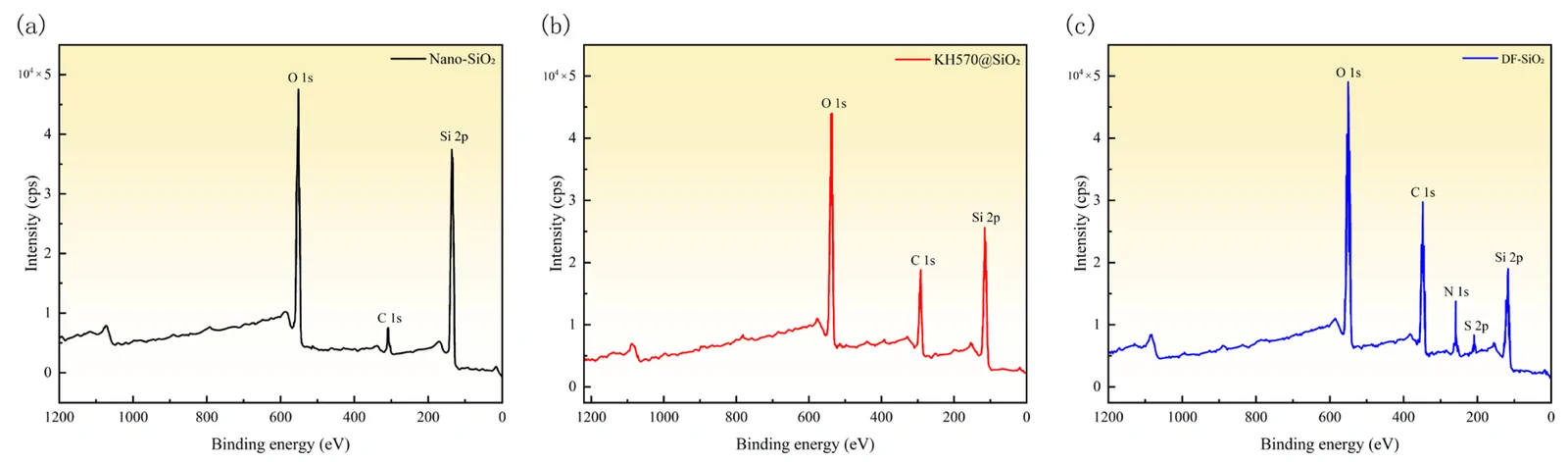
XPS spectra of different nano-SiO_2_ samples: (**a**) Nano‑SiO_2_; (**b**) KH570@SiO_2_; (**c**) DF‑SiO_2_

Figure 3 shows the full XPS spectra of Nano-SiO_2_, KH570@SiO_2_ and KH570@SiO_2_-AMPS/ODMA (DF-SiO_2_). The characteristic signals of Si 2p and O 1s can be observed in all three samples, indicating that the SiO_2_ inorganic skeleton structure can be maintained during the functionalization process. The spectrum of Nano-SiO_2_ is mainly composed of Si and O element signals, and a weak C 1s signal can be observed near about 285 eV, which can be attributed to the environmental carbon adsorbed on the surface of the sample. After KH570 modification, the C 1s signal was significantly enhanced, indicating that the organic components on the surface of the particles increased, which was consistent with the introduction of the organic silane structure in the KH570 molecule [25].

After further functionalization of KH570@SiO_2_ with AMPS/ODMA, the XPS full spectrum of DF-SiO_2_ showed obvious N 1s and S 2p signals in addition to Si, O and C elements. Among them, the N 1s signal is located near about 400 eV, and the S 2p signal is located in the range of about 168–170 eV. Since the AMPS molecule contains both amide nitrogen and sulfonic acid sulfur, while Nano-SiO_2_ and KH570@SiO_2_ do not have the corresponding structural N and S elements, the appearance of N 1s and S 2p signals in DF-SiO_2_ provides direct surface element evidence for the introduction of nano-SiO_2_ surface by AMPS-related functional structure [22,25]. At the same time, the C 1s signal of DF-SiO_2_ is further changed, which is consistent with the further introduction of AMPS/ODMA organic functional structure.

Combined with the results of FT-IR and TGA, it can be found that the organic structure and elemental composition of the material surface show continuous changes as SiO_2_ is bridged by KH570 and further functionalized by AMPS/ODMA. FT-IR reflects the change in characteristic functional groups, TGA indicates that the surface organic components are further increased, and XPS provides supplementary evidence from the perspective of surface element composition. The three characterization results confirm each other, indicating that KH570@SiO_2_-AMPS/ODMA has formed an organic surface layer containing AMPS/ODMA functional structure, which provides a material basis for the subsequent interfacial interaction between nanoparticles and HPAM and the construction of HPAM/DF-SiO_2_ composite gel system.

#### 2.1.4. DLS and Zeta Potential Characterization

**Figure 4:**
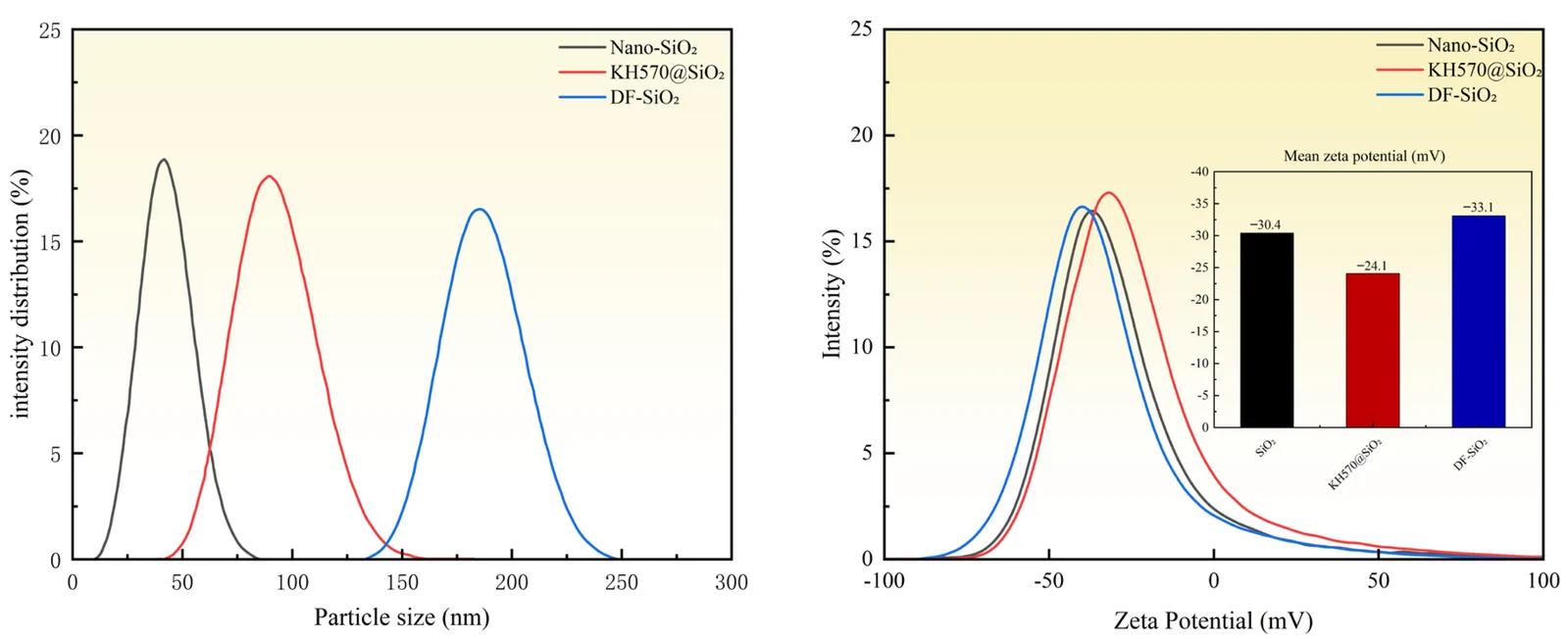
DLS particle size distribution and Zeta potential of different nano-SiO_2_ samples.

Figure 4 shows the DLS particle size distribution and Zeta potential results of Nano-SiO_2_, KH570@SiO_2_ and DF-SiO_2_. The average hydrodynamic particle size of Nano-SiO_2_ was about 42 nm, which increased to about 90 nm after KH570 modification. After further grafting AMPS/ODMA, the average hydrodynamic particle size of DF-SiO_2_ increased to about 185 nm. The three samples showed a relatively concentrated unimodal particle size distribution, and no obvious large-scale aggregation peaks were observed, indicating that the surface modification at each stage did not cause serious agglomeration of the particles in the test medium. The gradual increase in hydrodynamic particle size is related to the introduction of surface organic layer and the change in interfacial hydration layer. Among them, KH570 first forms an interface modification layer, and after the further introduction of the AMPS/ODMA functional segment, the organic layer and hydration structure on the surface of the particles are further thickened, thus showing a larger hydrodynamic size.

Zeta potential results show that the surface potentials of Nano-SiO_2_, KH570@SiO_2_ and DF-SiO_2_ are −30.4, −24.1 and −33.1 mV, respectively. After KH570 modification, some surface hydroxyl groups were replaced by organic silane structure, which weakened the negative charge of the particle surface [19,26]. After further grafting of AMPS/ODMA, the Zeta potential of DF-SiO_2_ was restored and further reduced to −33.1 mV, which was mainly related to the introduction of sulfonic acid groups in AMPS [27,28]. The higher negative potential is beneficial to enhance the electrostatic repulsion between particles and reduce the aggregation tendency in the aqueous phase. Combined with the DLS particle size distribution, it can be seen that although DF-SiO_2_ exhibits a larger hydrodynamic size due to the introduction of the functional layer, it still maintains a relatively stable dispersion state.

The above results indicate that DF-SiO_2_ still has good interfacial dispersion characteristics after multi-step surface functionalization, and the introduced AMPS and ODMA functional structures on its surface provide available action sites for the subsequent interfacial association, hydrophobic association and dynamic network construction of HPAM segments. Therefore, FT-IR, TGA and DLS/Zeta results together constitute the structural basis of DF-SiO_2_ participating in the construction of composite gel system.

### 2.2. Construction and Properties of HPAM/DF-SiO_2_ Composite Gel System

#### 2.2.1. Composition Screening of HPAM/DF-SiO_2_ Composite Gel System

**Figure 5:**
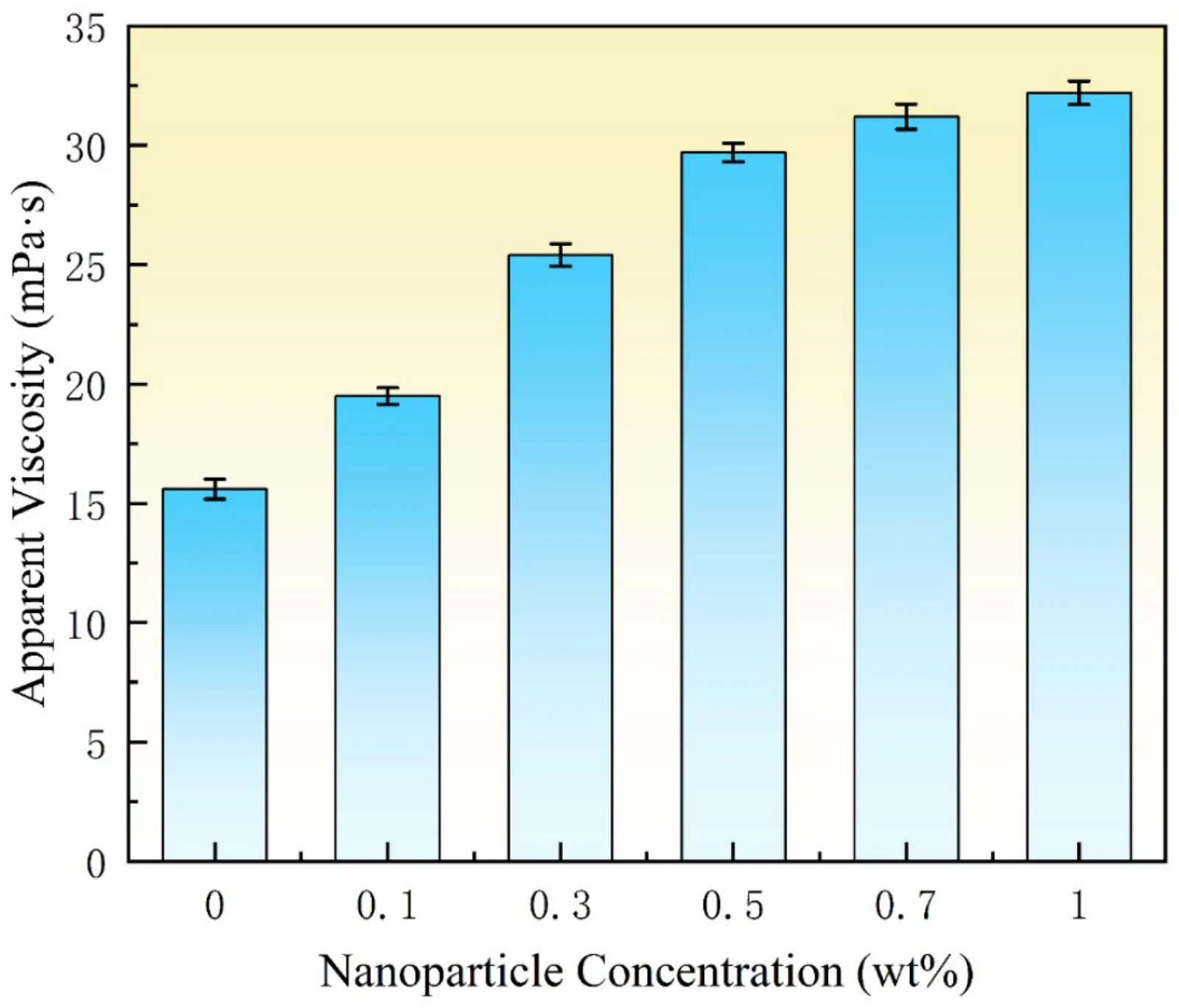
Effect of DF-SiO_2_ addition on the apparent viscosity of the composite gel system.

Based on the previous concentration screening results, the mobility control requirements of subsequent displacement experiments and the comprehensive consideration of polymer dosage, 1500 mg/L was selected as the working concentration of HPAM, and the effect of DF-SiO_2_ addition on apparent viscosity was investigated at 90 °C and 20,000 mg/L salinity. The results are shown in Figure 5. The viscosity of HPAM system without DF-SiO_2_ was 15.6 mPa·s. After adding DF-SiO_2_, the viscosity of the system increased with the increase in the addition amount. When the addition amount was 0.5 wt%, the viscosity of the system reached 29.7 mPa·s, which was 14.1 mPa·s higher than that of pure HPAM. When the addition amount increased to 1.0 wt%, the viscosity was 32.2 mPa·s, which was only 2.5 mPa·s higher than that of 0.5 wt%, and the viscosity increase was significantly slowed down.

At a lower addition amount, the functional segments and polar groups on the surface of DF-SiO_2_ can increase the interfacial correlation between nanoparticles and HPAM molecular chains, so that more polymer segments participate in spatial correlation, thus promoting the construction of internal network structure of the system [15,17]. As the content of DF-SiO_2_ continues to increase, the available interface interaction sites gradually become saturated, and the contribution of further increasing nanoparticles to the overall structure of the system is limited, so the increase in viscosity gradually decreases. Based on the enhancement effect of the system structure and the subsequent performance evaluation and economic evaluation results, 0.5 wt% DF-SiO_2_ was finally selected as the addition amount of the subsequent HPAM/DF-SiO_2_ composite gel system.

#### 2.2.2. Evaluation of Dispersion Stability of HPAM/DF-SiO_2_ Composite Gel System

On the basis of the above screening of HPAM/DF-SiO_2_ composite gel system, in order to further investigate the effect of different amounts of DF-SiO_2_ on the dispersion stability of the system, the concentration of HPAM was selected as 1500 mg/L, and the sealed standing experiment was carried out under the condition of 20,000 mg/L simulated formation water and 90 °C. The short-term dispersion stability of the system was characterized by measuring the relative concentration C/C_0_ of the dispersed DF-SiO_2_ in the supernatant at different standing time, where C is the concentration of DF-SiO_2_ in the supernatant at a specific standing time, and C_0_ is the initial concentration. Three parallel tests were performed in each experiment, and the results were expressed as mean ± standard deviation.

**Figure 6:**
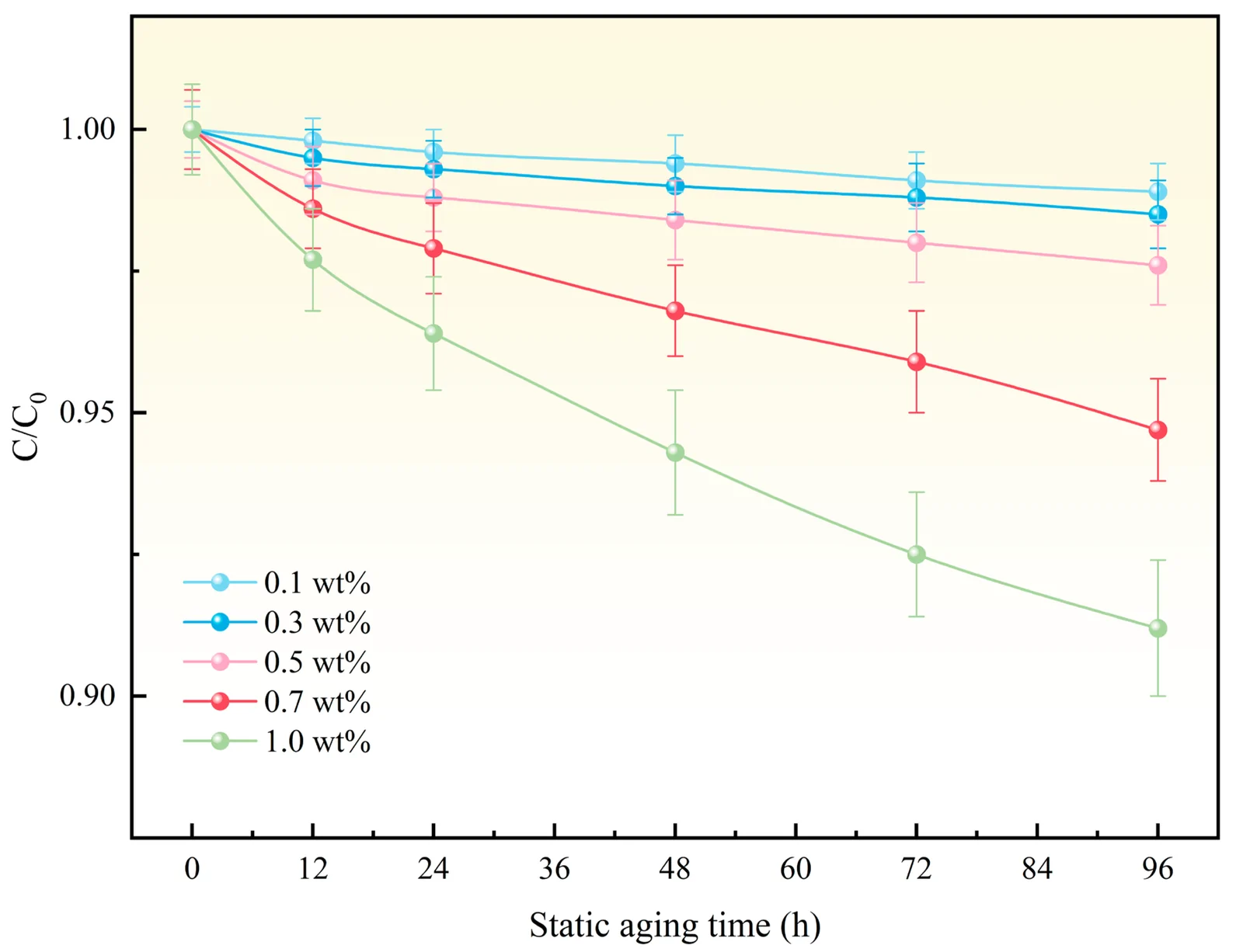
Short-term dispersion stability of HPAM/DF-SiO_2_ composite gel system with different amounts of DF-SiO_2_.

As shown in Figure 6, the C/C_0_ of each system gradually decreased with the extension of standing time, indicating that under the condition of high temperature and high salinity, the nanoparticles in the system would settle or disperse to a certain extent with the increase in standing time. There are obvious differences in the variation range of different DF-SiO_2_ addition systems. The C/C_0_ of 0.1 wt%, 0.3 wt% and 0.5 wt% DF-SiO_2_ systems remained at a high level throughout the test period, and the C/C_0_ of 0.5 wt% DF-SiO_2_ system was still about 0.97 after standing for 96 h, indicating that it had good short-term dispersion stability during the test period [12,26]. As the amount of DF-SiO_2_ added further increased to 0.7 wt% and 1.0 wt%, the decrease in C/C_0_ gradually increased, indicating that the higher amount of nanoparticles added further changed the dispersion state of the system and showed a more obvious tendency to settle.

With the increase in DF-SiO_2_ addition, the interaction between particles is enhanced, and the probability of effective collision of particles in the system increases. Therefore, it is more likely to have a certain degree of aggregation and sedimentation at a higher addition amount, resulting in a gradual decrease in the concentration of dispersed particles in the supernatant [26,28]. It should be noted that this experiment is mainly used to compare the short-term dispersion stability of the system under different DF-SiO_2_ additions. The test period is 96 h, and the results are not used to characterize the long-term storage stability of the material.

Combined with the above system composition screening results, it can be seen that the increase in DF-SiO_2_ addition is beneficial to further improve the viscosity of the system, but when the addition amount continues to increase, the short-term dispersion stability of the system gradually decreases. For the subsequent displacement experiments, 0.5 wt% DF-SiO_2_ can meet the requirements of the system’s fluidity control performance, while maintaining good short-term dispersion stability at 90 °C and high-salinity conditions. Therefore, it is not necessary to pursue higher system viscosity simply by further increasing the content of nanoparticles. Considering the performance requirements, dispersion stability and material dosage of the system, 0.5 wt% DF-SiO_2_ was finally selected as the working addition amount of the subsequent HPAM/DF-SiO_2_ composite gel system.

#### 2.2.3. Viscoelastic Characteristics of HPAM/DF-SiO_2_ Composite Gel System

**Figure 7:**
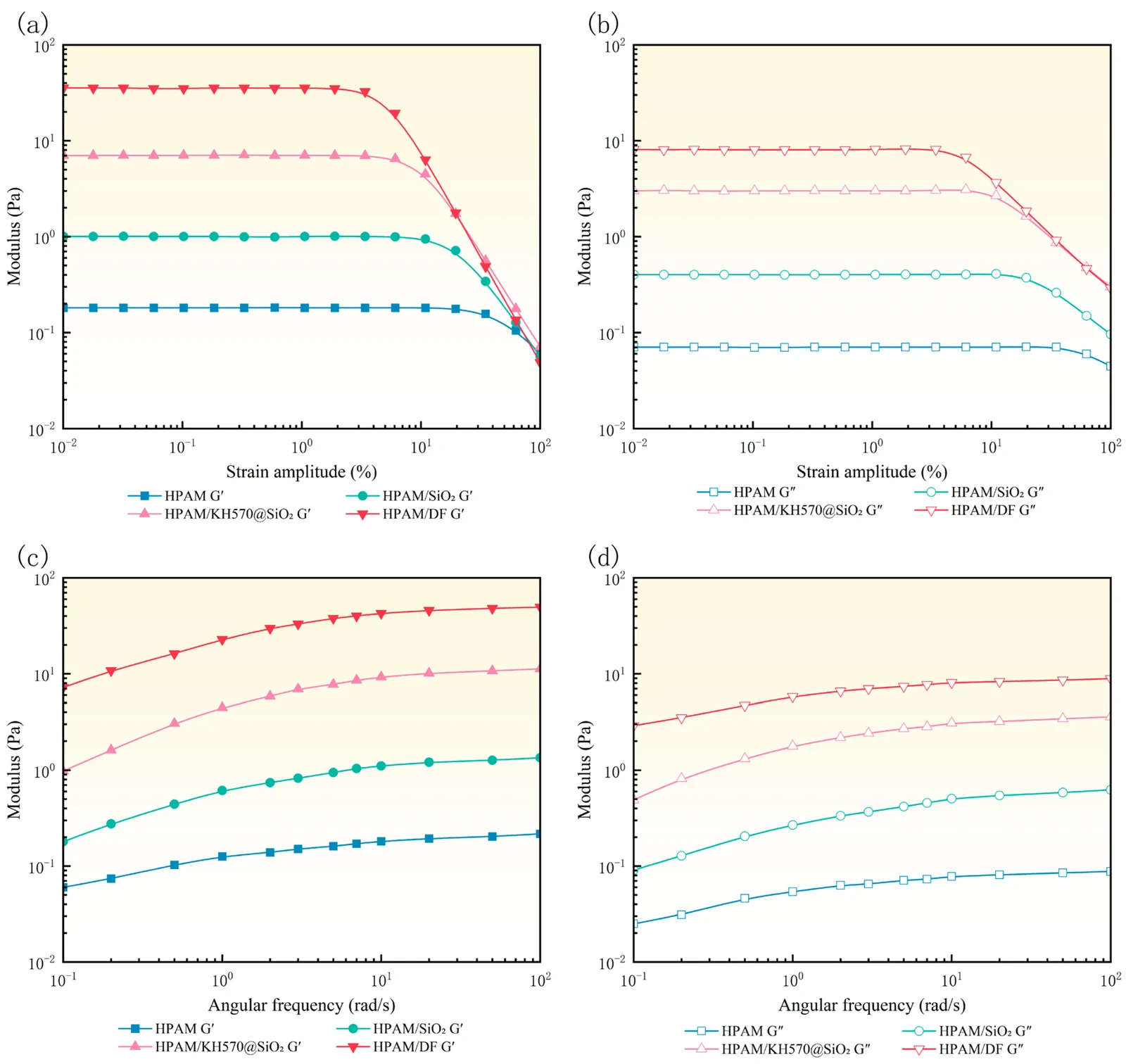
Viscoelastic response of different composite gel systems: (**a**) Storage modulus vs strain amplitude; (**b**) Loss modulus vs strain amplitude; (**c**) Storage modulus vs angular frequency; (**d**) Loss modulus vs angular frequency.

Figure 7 shows the results of strain scanning and oscillation frequency scanning of different HPAM composite systems. Firstly, the linear viscoelastic region (LVR) of the system was determined by strain scanning at 90 °C and angular frequency of 10 rad·s^−1^. As shown in Figure 7a,b the storage modulus (G′) and loss modulus (G″) of each system remain relatively stable in the low strain range, indicating that the system can maintain a stable viscoelastic structure under small strain perturbations. As the strain further increases, the G′ and G″ of each system decrease to varying degrees, indicating that the larger strain disturbance will destroy the internal correlation structure of the system. The 1% strain is in the platform area where the modulus of each system is basically stable, so 1% is selected as the fixed strain for subsequent oscillation frequency scanning.

Under the condition of 1% fixed strain, the oscillation frequency scanning of 0.1–100 rad·s^−1^ is further carried out. As shown in Figure 7c,d, in the whole test frequency range, the G′ of each system is higher than G″, indicating that the whole system exhibits viscoelastic characteristics dominated by elastic response. With the increase in angular frequency, G′ and G″ show an increasing trend as a whole, and the change range in the high frequency region gradually decreases, indicating that the viscoelastic response of the system tends to be gentle with the change in frequency. In order to further quantitatively characterize the frequency dependence of the system, different frequency intervals were compared. According to the linearity of the double logarithmic relationship, the number of data points and the requirement of the unified fitting interval for each system, the data in the range of 1–10 rad·s^−1^ were selected for power-law fitting. The interval contains 1, 2, 3, 5, 7 and 10 rad·s^−1^, a total of 6 data points. The storage modulus and loss modulus were described by $G^{\prime }=A\omega^{n^{\prime }}$ and $G^{\prime \prime }=B\omega^{n^{\prime \prime }}$, respectively, and linear regression was performed after logarithmic linearization. The fitting parameters are shown in Table 1.

The eight groups of data showed good power-law fitting correlation, in which the R^2^ of G′ was 0.975–0.998, and the R^2^ of G″ was 0.982–0.997. Both n′ and n″ are positive, indicating that the elastic response and viscous response of the system have a certain frequency dependence in the selected frequency range. In order to facilitate quantitative comparison between different systems under unified test conditions, G′ and G″ under 100 rad·s^−1^ are further given as reference points.

At this time, the G′ of HPAM, HPAM/SiO_2_, HPAM/KH570@SiO_2_ and HPAM/DF-SiO_2_ are 0.217 Pa, 1.35 Pa, 11.25 Pa and 49.6 Pa, respectively, and the corresponding G″ are 0.088 Pa, 0.625 Pa, 3.58 Pa and 8.90 Pa, respectively.

With the increase in the surface functionalization degree of nano-SiO_2_, the viscoelastic response of the composite system is gradually enhanced. After the addition of HPAM to unmodified SiO_2_, both G′ and G″ increased, indicating that the introduction of nanoparticles enhanced the viscoelastic response of the system, and the interfacial interaction between the surface of the nanoparticles and the HPAM segment was involved in the structural correlation within the system [15,17]. After KH570 modification, the G′ of HPAM/KH570@SiO_2_ system was further increased, indicating that KH570 modification enhanced the interfacial association between nanoparticles and HPAM segments and promoted the formation and stability of the internal structure of the system [20]. After further introducing the AMPS/ODMA functional segment, the HPAM/DF-SiO_2_ system exhibits a higher elastic response, and its G′ at 100 rad·s^−1^ reaches 49.6 Pa, which is about 4.4 times that of the HPAM/KH570@SiO_2_ system, reflecting that the functionalized nanoparticles enhance the structural correlation within the system.

Combined with the power-law fitting results, the n′ and n″ of different systems showed a certain frequency dependence. The n′ of HPAM/DF-SiO_2_ is 0.269, which is lower than 0.315 of HPAM/KH570@SiO_2_, indicating that the elastic response of the former is less sensitive to frequency changes. Combined with its high G′ in the whole test frequency range, it shows that the HPAM/DF-SiO_2_ system has a strong and relatively stable elastic response.

This enhancement corresponds to the bifunctional structure of the DF-SiO_2_ surface. The sulfonic acid group contained in AMPS helps to improve the hydrophilicity of the functional layer and the interface stability in the salt environment [23,24], and the long chain of ODMA enhances the dynamic association between polymer segments through hydrophobic association [17,18,19]. Therefore, the role of DF-SiO_2_ in the HPAM system is not only reflected in the enhanced filling of nanoparticles, but also through the nanoparticle–polymer interface interaction and hydrophobic association to participate in the internal structure construction of the system, so that the HPAM/DF-SiO_2_ system exhibits a stronger elastic response. The higher G′ reflects that the system has stronger structural support ability under small amplitude oscillation conditions, and also provides a rheological basis for the subsequent investigation of its stability under a high-temperature and high-salt environment and the structural recovery behavior after shear disturbance.

#### 2.2.4. Temperature Stability of HPAM/DF-SiO_2_ Composite Gel System

**Figure 8:**
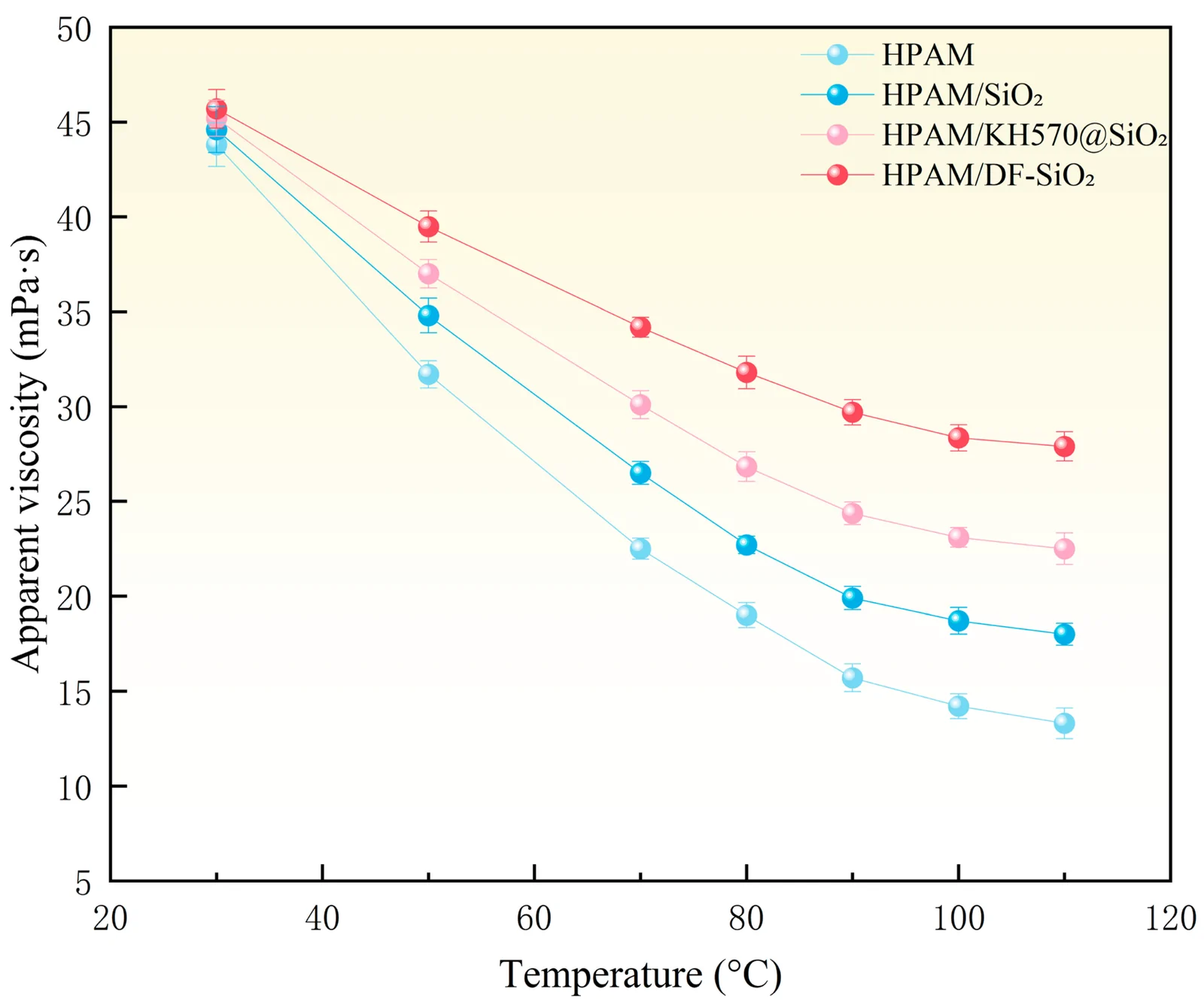
Temperature–viscosity relationship of different composite gel systems.

When the temperature increases from 30 °C to 110 °C, the apparent viscosity of each system decreases gradually, but the viscosity retention ability of different systems is different, as shown in Figure 8. The apparent viscosity of HPAM decreased from 43.8 mPa·s at 30 °C to 13.3 mPa·s at 110 °C. After the introduction of SiO_2_ and further surface modification, the viscosity decreased significantly. At 90 °C, the apparent viscosities of HPAM, HPAM/SiO_2_, HPAM/KH570@SiO_2_ and HPAM/DF-SiO_2_ were 15.7, 19.9, 24.4 and 29.7 mPa·s, respectively. Compared with HPAM, the apparent viscosity of HPAM/DF-SiO_2_ was increased by about 89.2%, which was about 49.2% and 21.7% higher than that of HPAM/SiO_2_ and HPAM/KH570@SiO_2_, respectively. At 110 °C, HPAM/DF-SiO_2_ still maintained 27.9 mPa·s, which was the highest value in each system. The above results show that the HPAM/DF-SiO_2_ composite gel system maintains the highest viscosity during the whole heating process, showing slower structural decay characteristics.

For the HPAM system, the increase in temperature will enhance the thermal motion of the polymer chain segment and weaken the intermolecular interaction, so that the original chain entanglement structure is gradually relaxed, resulting in a decrease in the viscosity of the system [2]. In contrast, the interfacial interaction on the surface of SiO_2_ particles can limit the HPAM segment and slow down the relaxation of the polymer network structure at high temperature [13,20]. After further functionalization with KH570 and AMPS/ODMA, the interfacial association between nanoparticles and HPAM was enhanced, so that the dynamic composite network could maintain more effective structural connections during the heating process. When the temperature increased from 50 °C to 90 °C, the viscosity of HPAM decreased from 31.7 mPa·s to 15.7 mPa·s, while that of HPAM/DF-SiO_2_ decreased from 39.5 mPa·s to 29.7 mPa·s.

When the temperature is further increased to 110 °C, HPAM/DF-SiO_2_ still maintains 27.9 mPa·s, which is about 2.1 times that of HPAM system 13.3 mPa·s. It can be seen that DF-SiO_2_ does not eliminate the viscosity attenuation caused by the increase in temperature, but reduces the structural loss of the system under high-temperature conditions by maintaining the nanoparticle–polymer interface interaction and dynamic network structure, so that the composite gel exhibits better temperature stability.

#### 2.2.5. Salt Tolerance Stability of HPAM/DF-SiO_2_ Composite Gel System

**Figure 9:**
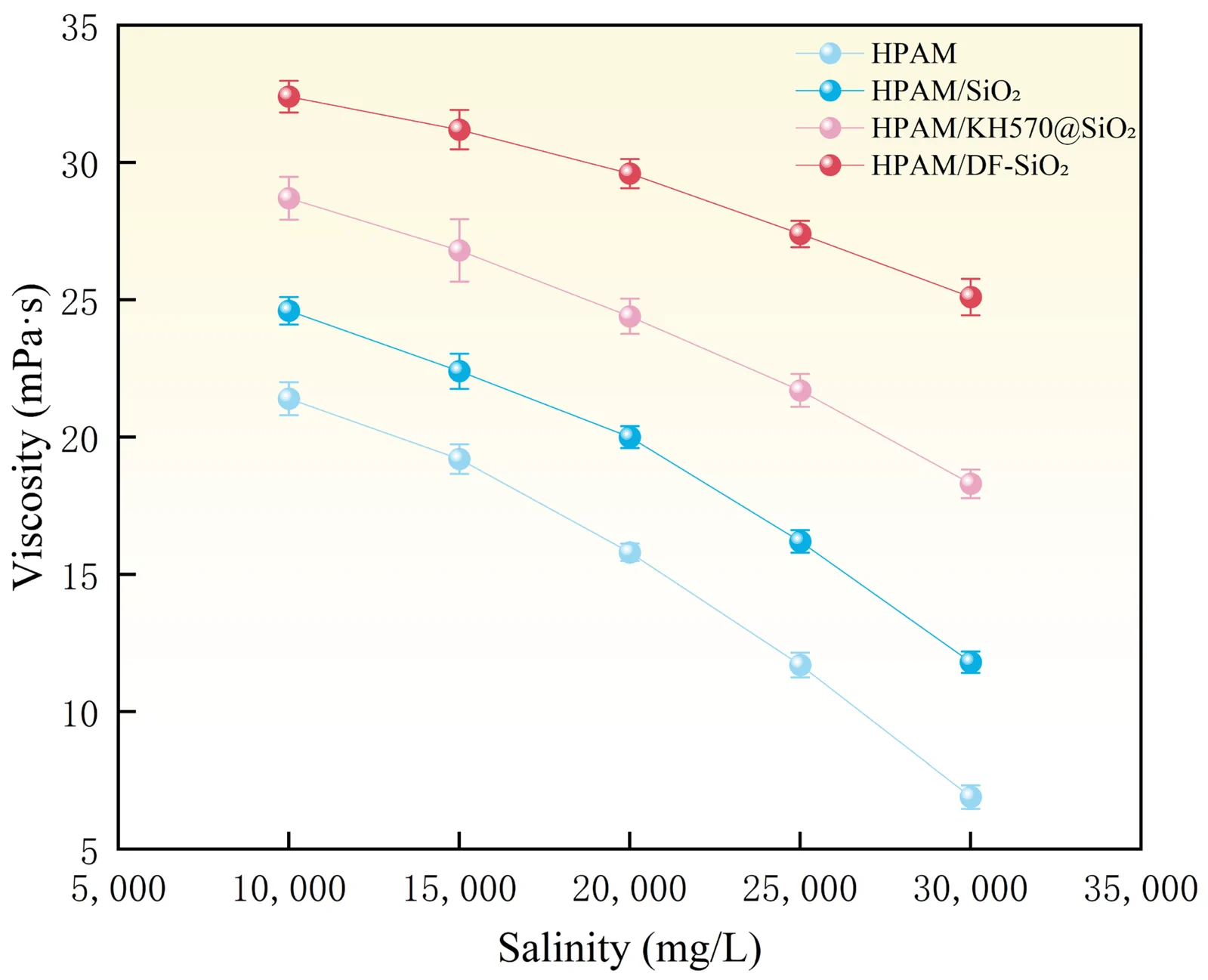
The relationship between salinity and viscosity of different composite gel systems.

With the increase in salinity, the viscosity of each system gradually decreased, but the salt sensitivity of different gel systems was significantly different. Figure 9 shows the apparent viscosity changes of different HPAM composite gel systems in the range of 10,000–30,000 mg/L salinity at 90 °C. The apparent viscosity of HPAM was 21.4 mPa·s at 10,000 mg/L, and increased to 24.6 mPa·s after adding SiO_2_. The apparent viscosity of HPAM/KH570@SiO_2_ and HPAM/DF-SiO_2_ reached 28.7 and 32.4 mPa·s, respectively. After the increase in salinity, the viscosity of HPAM decreased obviously, while the decrease in the composite system was relatively small, and the improvement of functionalized SiO_2_ was more prominent. When the salinity reaches 30,000 mg/L, HPAM/DF-SiO_2_ still maintains a high apparent viscosity, and its viscosity retention rate reaches 77.5%, indicating that the composite gel system can reduce the viscosity loss under high-salinity conditions.

HPAM is more sensitive to salinity changes, mainly due to the electrostatic shielding effect produced by salt ions, which weakens the repulsion between polymer chains, makes the molecular chain conformation tend to shrink, and then leads to the decrease in rheological properties of the system [20,29,30]. For the composite gel system, the introduction of SiO_2_ can provide additional interfacial interaction and change the spatial correlation state of the polymer chains in the aqueous phase. After functionalization, the interaction between nanoparticles and polymers is further enhanced, thereby alleviating the adverse effect of salt environment on the viscosity of the system [17,23,27]. Among them, the sulfonic acid group introduced by AMPS can improve the hydrophilicity and ion tolerance of the functional layer, which is helpful to maintain the interface stability under high-salinity conditions [18,23,24]; the long chain of ODMA can enhance the dynamic association between polymer chains through hydrophobic association, providing a certain reversible physical connection for the composite gel network [17,18,19]. Therefore, with the increase in salinity, HPAM/DF-SiO_2_ can still maintain a relatively stable gel network correlation, and its performance degradation degree is significantly lower than that of the unmodified HPAM system.

On the whole, the salt tolerance of DF-SiO_2_ is not simply manifested as the increase in viscosity, but the salt-tolerant structure of AMPS and the dynamic hydrophobic association of ODMA participate in the stable maintenance of the composite gel network, so that the system still has good structure retention ability under high-salinity conditions.

#### 2.2.6. Structural Recovery Behavior of HPAM/DF-SiO_2_ Composite Gel System

**Figure 10:**
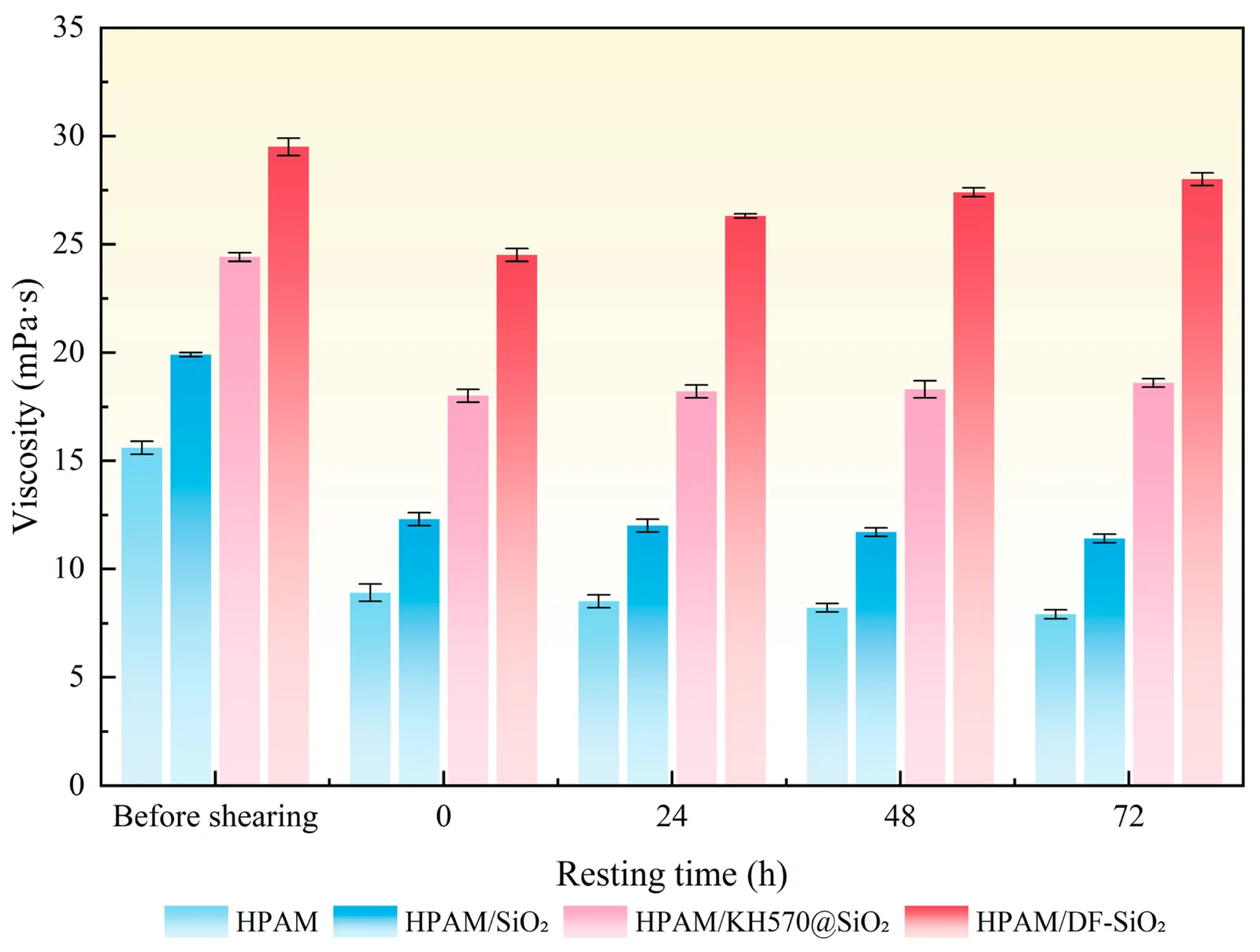
Viscosity-recovery characteristics of different composite gel systems after shearing.

Figure 10 shows the viscosity change and standing recovery process of different composite gel systems after strong shear. After strong shear, the viscosity of each system decreased significantly, but the degree of recovery was different. The viscosity of the HPAM system continued to decrease after shearing, and did not return to the pre-shear level after standing for 72 h. The change rule of HPAM/SiO_2_ system is similar to that of HPAM system, indicating that the introduction of SiO_2_ can improve the initial viscosity of the system, but the improvement of viscosity recovery after shearing is not obvious. The viscosity of HPAM/KH570@SiO_2_ system decreased slightly after shearing, and then remained stable, showing good structure retention ability. The HPAM/DF-SiO_2_ system showed a significant recovery process, and the viscosity gradually increased with the extension of standing time after shearing, and it was close to the pre-shear level after 72 h.

**Figure 11:**
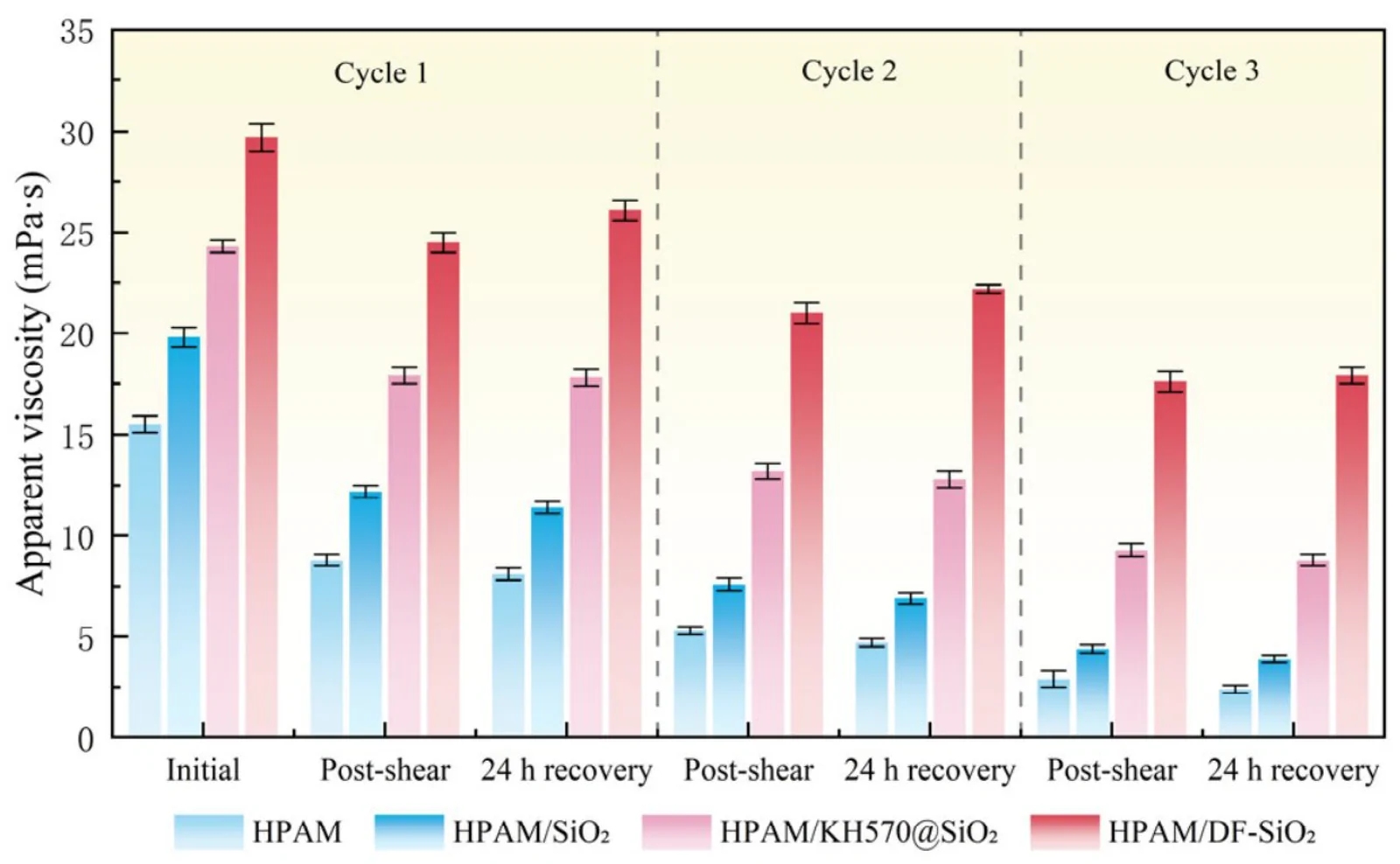
Multiple shear-recovery behavior of different composite gel systems.

In order to further investigate the change in this recovery behavior under repeated shear, three shear-recovery cycles were performed on each system, and the results are shown in Figure 11. As the number of cycles increases, the viscosity of the four systems after shearing is further reduced, but the performance of the recovery phase is different. HPAM and HPAM/SiO_2_ systems did not show obvious viscosity recovery after each cycle of recovery. The viscosity of HPAM/KH570@SiO_2_ system changed little before and after recovery, which mainly showed a certain structure retention effect. In contrast, the viscosity of the HPAM/DF-SiO_2_ system recovered from 24.5 mPa·s, 21.0 mPa·s and 17.6 mPa·s to 25.6 mPa·s, 21.5 mPa·s and 17.8 mPa·s after three cycles, respectively. Although the recovery amplitude decreases with the increase in the number of cycles, a certain degree of viscosity recovery can be observed after each shear.

Strong shear will destroy the entanglement of polymer chains and the physical correlation between particles and polymers, resulting in the relaxation of the internal structure of the system. After the shear stops, the hydrophobic association and the interfacial interaction between the nanoparticles and the polymer chain are re-established, and the viscosity of the system is restored [31,32,33,34,35,36,37,38]. For HPAM system, this kind of reversible physical effect is weak, and the structural loss is obvious after continuous shearing, so the recovery ability is limited. After the introduction of SiO_2_, the particle surface can form a certain physical correlation with the HPAM chain segment, and the structure retention ability of the system is improved. After KH570 modification, the interfacial bonding between the particles and the polymer was further enhanced, so the viscosity decrease after shearing was reduced [19,20]. On this basis, the introduction of AMPS/ODMA functional segments further strengthens the interfacial interaction between DF-SiO_2_ and HPAM, and increases the dynamic connection between polymer chains through the hydrophobic association provided by ODMA segments [17,18,19,23,24]. Therefore, the HPAM/DF-SiO_2_ system can maintain a certain viscosity recovery ability after single and multiple shearing, indicating that its internal physical correlation structure has good reversible reconstruction characteristics.

The HPAM/DF-SiO_2_ composite gel system showed a certain degree of viscosity recovery during continuous shear-recovery process, indicating that the internal physical correlation structure of the system had reversible reconstruction ability. As the number of cycles increases, the recovery amplitude gradually decreases, indicating that repeated shearing will cause certain cumulative structural damage, thus reducing the recovery degree of the system. However, the viscosity recovery can still be observed after three cycles, indicating that the dynamic physical correlation structure can still be continuously reconstructed. Compared with other systems, HPAM/DF-SiO_2_ shows more obvious repeated recovery characteristics, indicating that DF-SiO_2_ enhances the structure retention and recovery ability of the system under continuous shear disturbance.

#### 2.2.7. Long-Term Thermal Aging Stability of HPAM/DF-SiO_2_ Composite Gel System

**Figure 12:**
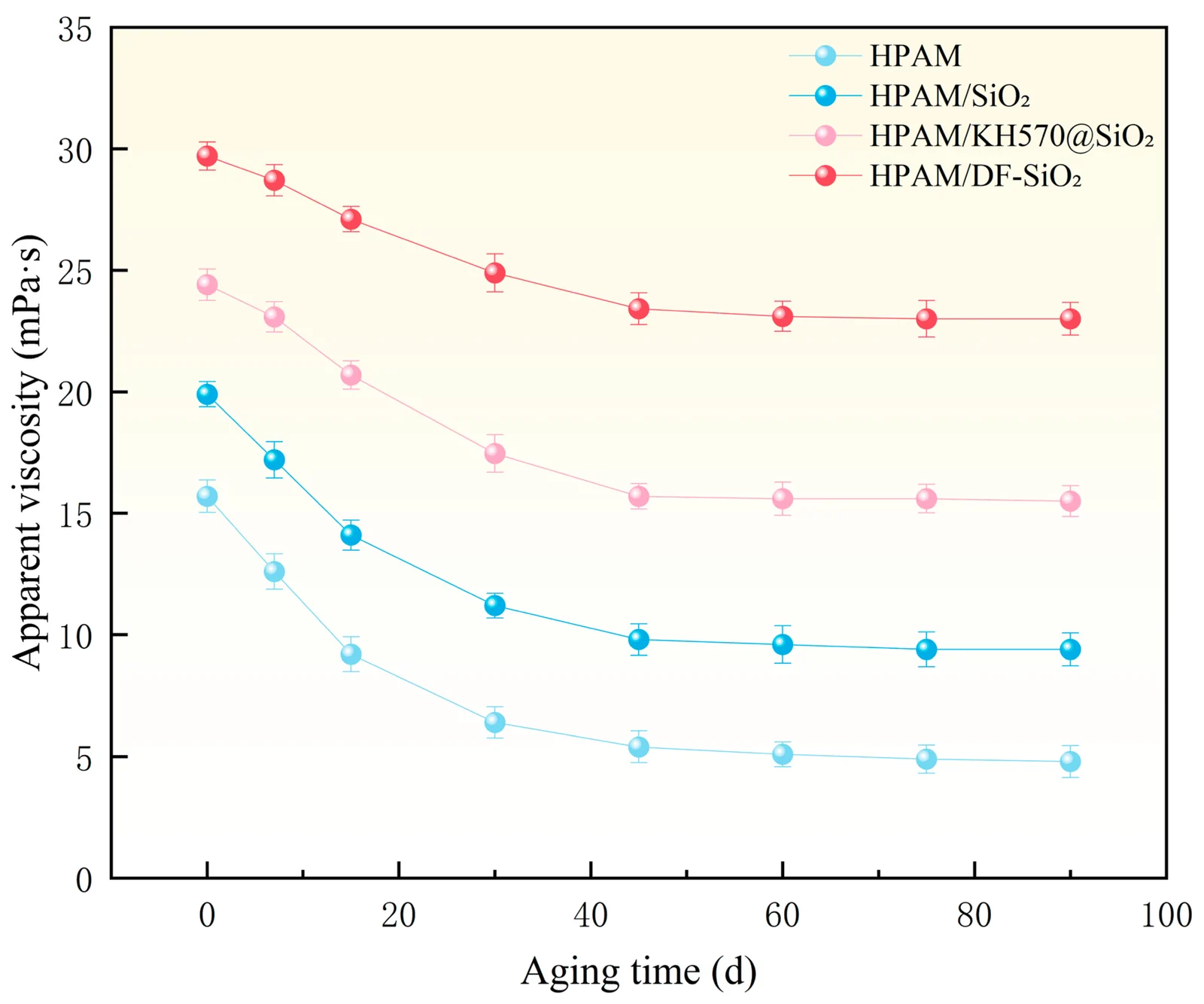
Viscosity changes of different composite gel systems during long-term thermal aging.

Figure 12 shows the apparent viscosity changes of different composite gel systems during aging at 90 °C for 90 days. The viscosity of each system decreased with the extension of aging time, but the degree of attenuation was significantly different. The HPAM system decreased rapidly from the initial 15.7 mPa·s to 6.4 mPa·s at 30 d, and further decreased to 4.8 mPa·s at 90 d. In contrast, the HPAM/SiO_2_ and HPAM/KH570@SiO_2_ systems maintained 9.4 and 15.5 mPa·s after 90 d, respectively. HPAM/DF-SiO_2_ showed the most stable aging characteristics, and its initial viscosity was 29.7 mPa·s, which remained 23.0 mPa·s after 90 days, and the overall decline was only 22.6%. It is worth noting that the viscosity change in the system slowed down significantly after 45 days, and only decreased by about 0.4 mPa·s in the following 45 days, showing a good long-term stable state.

Under the action of long-term high temperature, HPAM is prone to the enhancement of molecular chain thermal motion, conformation relaxation and structural degradation, which makes the effective network structure continue to decrease, resulting in a decrease in the viscosity of the system [39,40]. At the same time, the simulated formation water with a salinity of about 20,000 mg/L was used in the aging experiment in this study. The high ionic strength would compress the electric double layer around the polymer molecular chain, weaken the electrostatic repulsion between the charged segments, make the HPAM molecular chain more prone to curl and shrink, and lead to the weakening of its hydrodynamic volume and the effective interaction between the molecular chains. Salt ions such as Ca^2+^ and Mg^2+^ in simulated formation water may further affect the local ion environment and polymer chain conformation, but the specific degree of action is related to the type and concentration of ions and the structure of the polymer itself. Therefore, under the combined action of high temperature and high salinity, HPAM is more prone to network structure relaxation and viscosity attenuation.

SiO_2_ and its surface functional layer can increase the interfacial correlation in the polymer system and limit the excessive shrinkage and rearrangement of HPAM molecular chains to a certain extent. For the HPAM/DF-SiO_2_ system, the sulfonic acid group introduced by AMPS has strong hydrophilicity, which is beneficial to maintain the hydration state of the surface of the functionalized particles and improve the adaptability of the interface to the high ionic strength environment. At the same time, the hydrophobic association formed between the long chains of ODMA can provide reversible physical connection, which helps to maintain the integrity of the network structure after being disturbed by a high-temperature and salt ion environment. Therefore, DF-SiO_2_ may weaken the adverse effect of a high-temperature and high-salinity environment on the composite network structure through the synergistic effect of interfacial interaction, hydration stability and reversible hydrophobic association.

Compared with the long-term thermal stability results of the existing nanoparticle-reinforced HPAM system, the stability level of the HPAM/DF-SiO_2_ system can be further reflected. After aging at 90 °C for 96 h, the viscosity retention rate of nano-SiO_2_/HPAM system reported by Guo et al. was 38.8–40.7%, and the highest viscosity retention rate of 1.0 wt% SiO_2_ system was 40.7% [41]. The silica/HAHPAM hybrid system reported by Zhu et al. had a viscosity retention rate of 33.83% after 65 days of aging at 85 °C and TDS of 32,868 mg/L [42]. In addition, Taborda et al.’s research showed that surface-modified SiO_2_ could improve the thermal stability of HPAM system, and the viscosity loss of modified SiO_2_ system with better performance was about 22.9% after aging at 70 °C for 15 days, and the corresponding viscosity retention rate was about 77.1% [43]. In contrast, the HPAM/DF-SiO_2_ system in this study still maintained 77.5% of the initial apparent viscosity after aging at 90 °C for 90 days, showing a high long-term viscosity retention level. Further, the viscosity change of HPAM/DF-SiO_2_ system slowed down significantly after 45 days, reflecting that it had good viscosity retention ability in the later stage of long-term thermal aging. Based on the aging curve and the comparison results in the literature, the introduction of DF-SiO_2_ not only improves the initial viscosity of the system, but also maintains a high viscosity level of the composite gel in the long-term high-temperature and high-salinity environment, which is beneficial to its long-term application under high-temperature and high-salinity conditions.

#### 2.2.8. Microscopic Network Structure of HPAM/DF-SiO_2_ Composite Gel System

**Figure 13:**
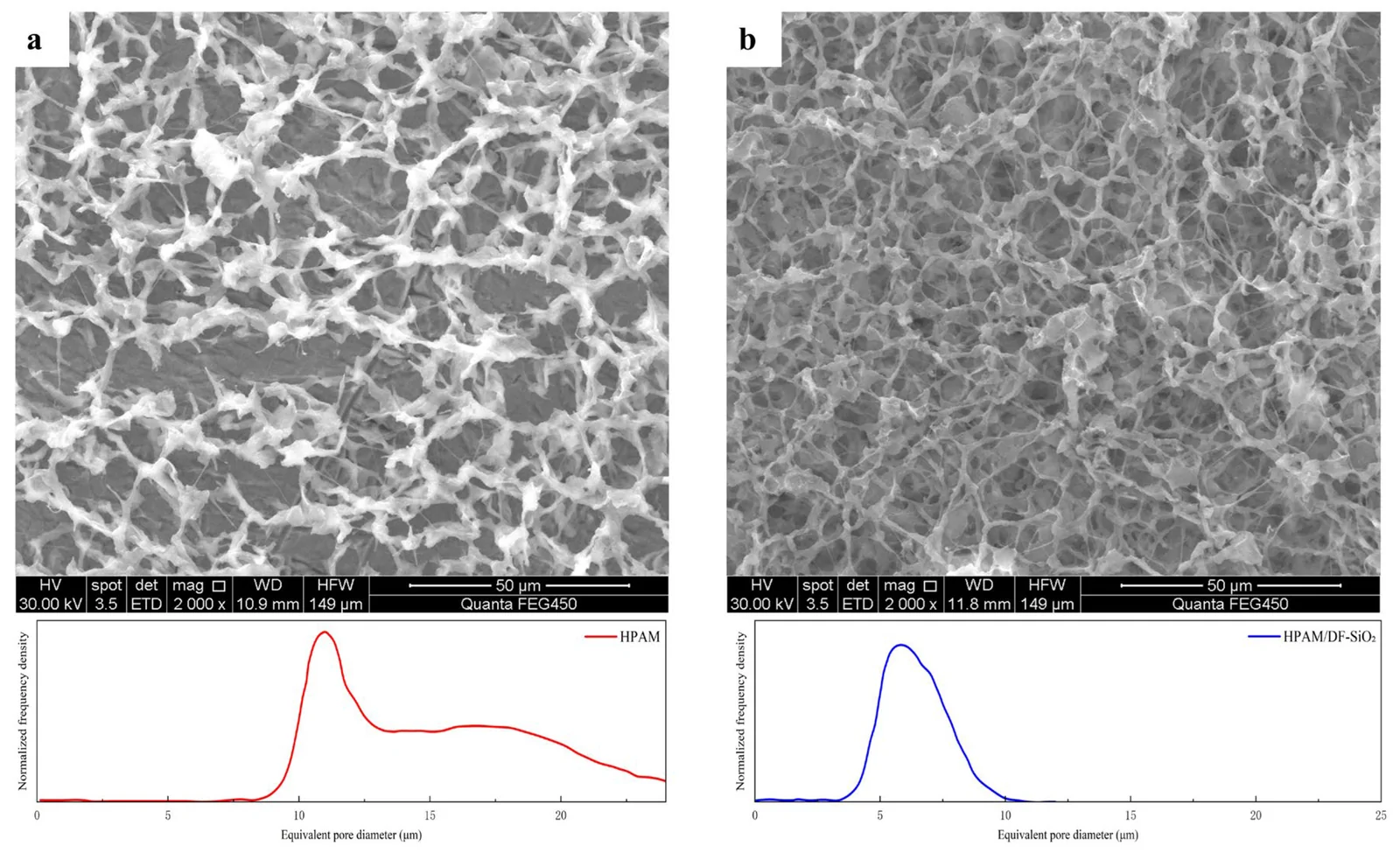
FESEM images and pore-size distribution of HPAM and HPAM/DF-SiO_2_ composite gel systems: (**a**) HPAM; (**b**) HPAM/DF-SiO_2_.

Figure 13 shows the FESEM images of HPAM and HPAM/DF-SiO_2_ systems after freeze-drying. Because the sample is freeze-dried, the FESEM image mainly reflects the spatial network morphology retained after dehydration of the system, which can be used to compare the microstructure, network continuity, and structural integrity of different systems, and cannot be directly equivalent to the gel network structure in the hydrated state.

As shown in Figure 13a, the pure HPAM system can form a certain three-dimensional network structure, but the overall network is relatively loose, the skeleton distribution is uneven, and there are large pores in the local area. From the microscopic morphology, the pore scale distribution is relatively wide, the pores in some areas are large, and the connection between the network skeletons is relatively loose. This is related to the fact that the HPAM molecular chain mainly relies on chain entanglement and intermolecular interaction to maintain the spatial structure. During the freeze-drying process, the water removal is accompanied by the shrinkage of the chain segment, so that the final retained network structure shows a certain degree of inhomogeneity [16,44,45].

After the introduction of DF-SiO_2_, the microscopic morphology of the system changes significantly, as shown in Figure 13b. Compared with pure HPAM, the HPAM/DF-SiO_2_ system showed a more continuous and uniform network structure, the connection between the network skeletons was more sufficient, the larger pores were significantly reduced, the pore space distribution tended to be uniform, and the continuity and compactness of the network skeleton were improved. Combined with the pore size distribution shown in Figure 13, it can be further seen that the pore size distribution of the HPAM/DF-SiO_2_ system moves to a smaller pore size range as a whole, and the distribution is more concentrated, indicating that the network pores inside the composite system are further refined. The above results indicate that DF-SiO_2_ participates in the construction of the HPAM composite network structure and enhances the spatial correlation between polymer chains, so that the system forms a denser and more continuous network structure. In similar nanoparticle/polymer composite gel systems, nano-SiO_2_ can be used as an interface node in the network to form a composite network with high structural integrity together with the polymer chain [28]. Relevant studies have also shown that the gel network structure and its pore space characteristics are closely related to the migration and distribution of the system in porous media, which provides a reference for understanding the structural correlation between the composite gel network and porous media [46].

**Figure 14:**
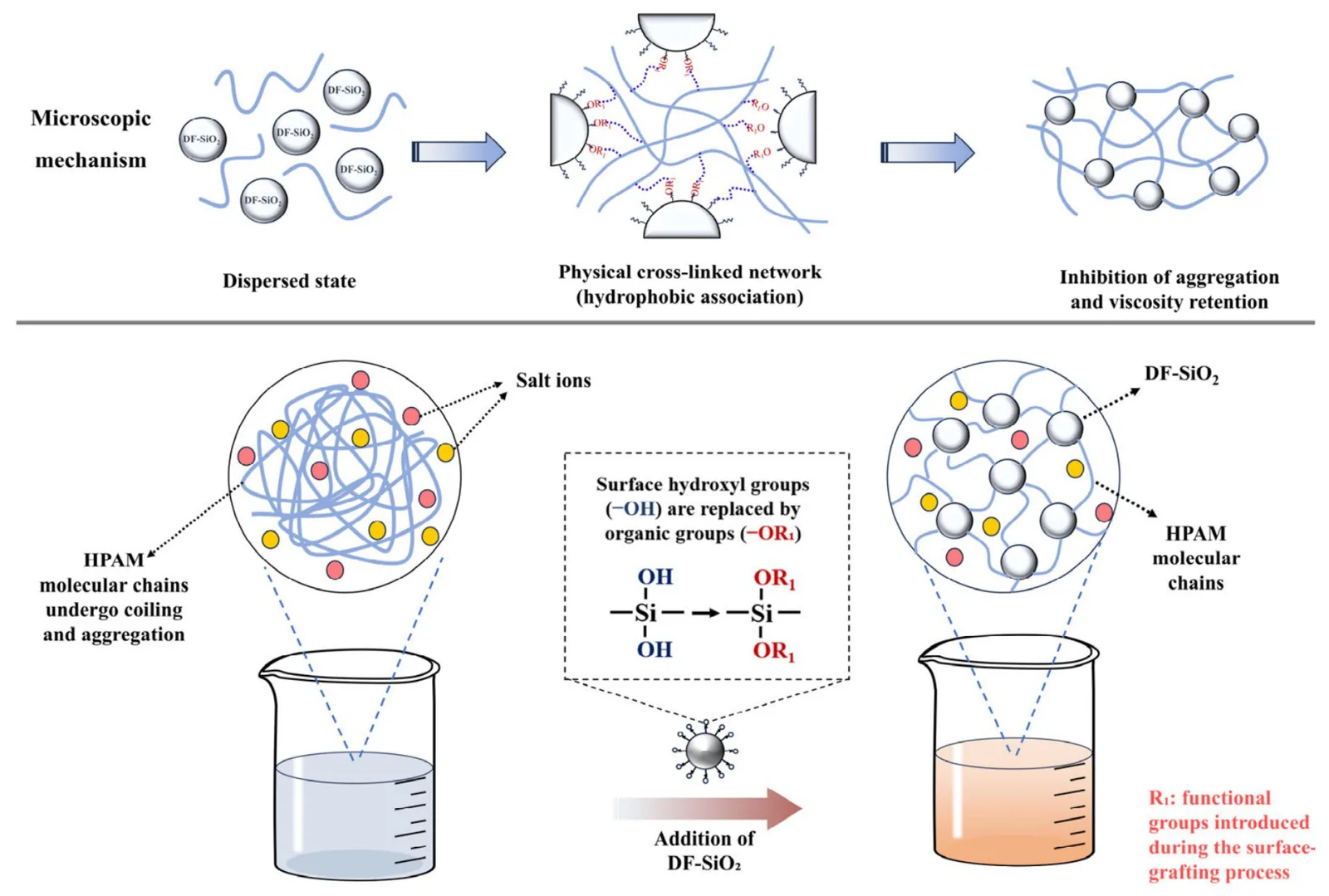
Network construction and mechanism of action of HPAM/DF-SiO_2_ composite gel system.

The microscopic process is shown in Figure 14. After HPAM chains contact with functionalized SiO_2_, a spatial correlation structure is formed under the action of nanoparticle interface and hydrophobic association. At the same time, the functionalized surface helps to improve the dispersion stability of particles in the system and maintain good continuity of the composite network. It can be seen from Figure 14 that DF-SiO_2_ is involved in the construction and stability of HPAM composite network. Among them, functionalized nanoparticles can be used as nodes between polymer chains to enhance network connectivity. The hydrophobic association provided by ODMA forms a reversible physical connection, which helps to maintain the network structure and promote the structural reconstruction after shear perturbation.

The above microstructure and network structure characteristics correspond to FT-IR, TGA, DLS/Zeta and rheological results, which support the participation of DF-SiO_2_ in the construction of HPAM composite gel network and its structural stabilization from different characterization levels. Therefore, the difference in network continuity and integrity presented by FESEM is consistent with the macroscopic rheology and viscosity recovery behavior after shearing, which indicates that DF-SiO_2_ can regulate the spatial network structure of HPAM composite gel through the synergistic effect of interface interaction and dynamic association, and help to maintain its structural integrity and structural reconstruction ability after shearing.

### 2.3. Study on the Structure Recovery of HPAM/DF-SiO_2_ Composite Gel System and Its Core Flow Control Performance

#### 2.3.1. Core Fluidity Experiment

**Figure 15:**
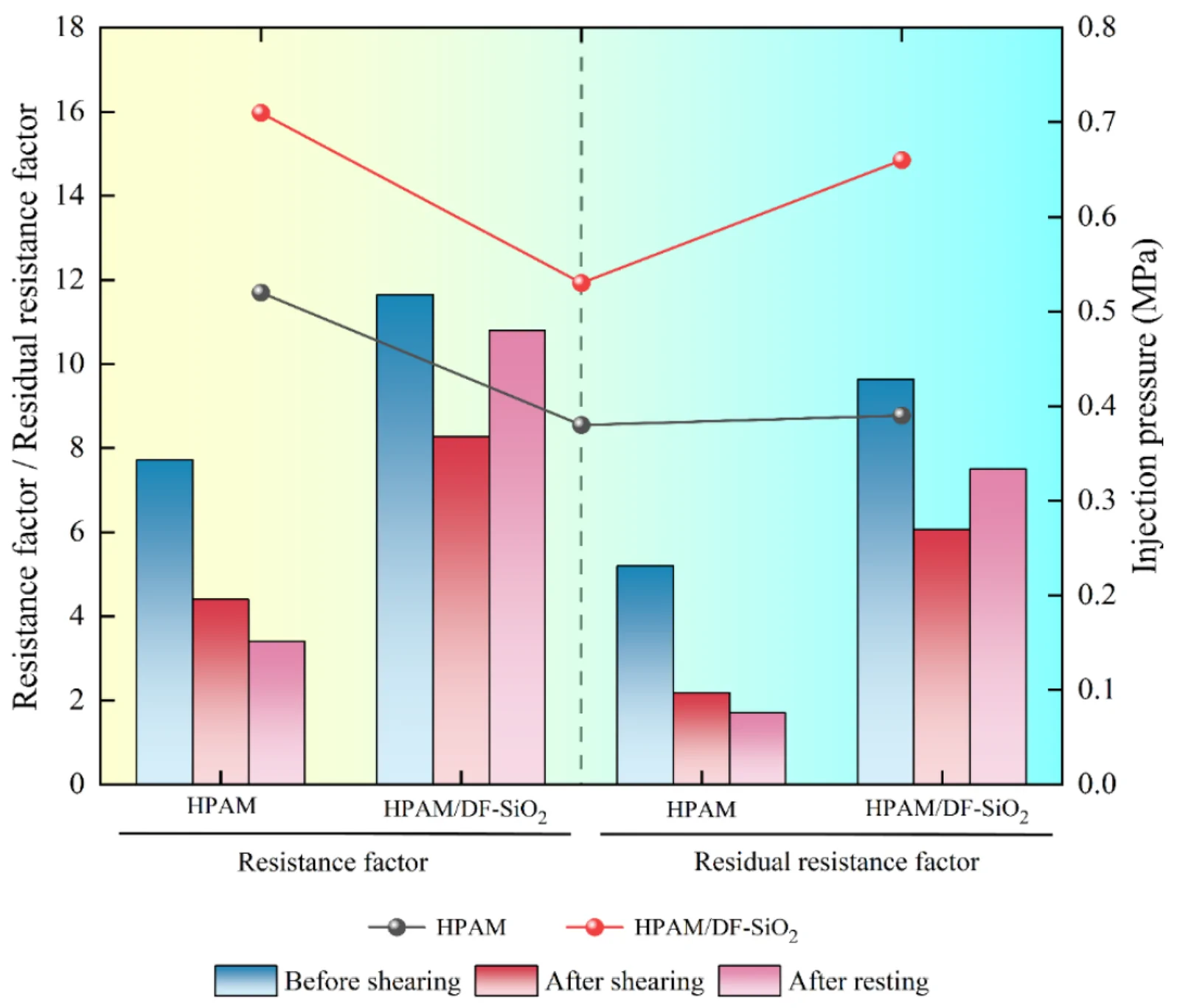
Core flow recovery characteristics of HPAM and HPAM/DF-SiO_2_ composite gel systems.

The change in core flow parameters further reflects the flow control ability of the composite gel system in porous media. As shown in Figure 15, the *RF* and *RRF* of HPAM/DF-SiO_2_ before shearing are 11.6 and 9.5, respectively, which are higher than 7.7 and 5.2 of HPAM system, indicating that the introduction of DF-SiO_2_ enhances the flow resistance and residual regulation of the system in core pores [47,48]. After shearing treatment, the *RF* of both systems decreased, among which HPAM decreased from 7.7 to 4.3, with a decrease of 44.2%, while HPAM/DF-SiO_2_ decreased from 11.6 to 8.2, with a decrease of 29.3%.

After standing for 5 days, the two systems showed significantly different seepage responses. The *RF* of HPAM was further reduced to 3.4, which was only 44.2% of that before shearing, while the *RF* of HPAM/DF-SiO_2_ recovered to 10.8, which was 93.1% of the initial value. *RRF* showed the same trend, DF-SiO_2_ system recovered to 7.4, while HPAM only recovered to 1.6. At the same time, the injection pressure of DF-SiO_2_ system decreased from 0.70 MPa to 0.53 MPa and then recovered to 0.65 MPa, while that of HPAM decreased from 0.52 MPa to 0.38 MPa.

The difference in recovery behavior between the two systems mainly comes from the difference in network reconfiguration ability. The HPAM chain entanglement is irreversibly damaged under high shear, so it is difficult to re-establish an effective seepage channel after standing [10,47,49]. However, the HPAM/DF-SiO_2_ composite gel system can still maintain a high flow resistance after experiencing the shear action in the porous medium, and restore part of the seepage control ability during the standing process. This corresponds to the structural reconstruction behavior observed in Section 2.2.6, indicating that the dynamic physical network constructed by DF-SiO_2_ still has certain reconfigurability after entering the porous medium, and can restore part of the network structure through the dynamic correlation between polymer chains after shear disturbance, thus maintaining good mobility control ability.

#### 2.3.2. Double-Tube Parallel-Core Displacement Experiment

**Figure 16:**
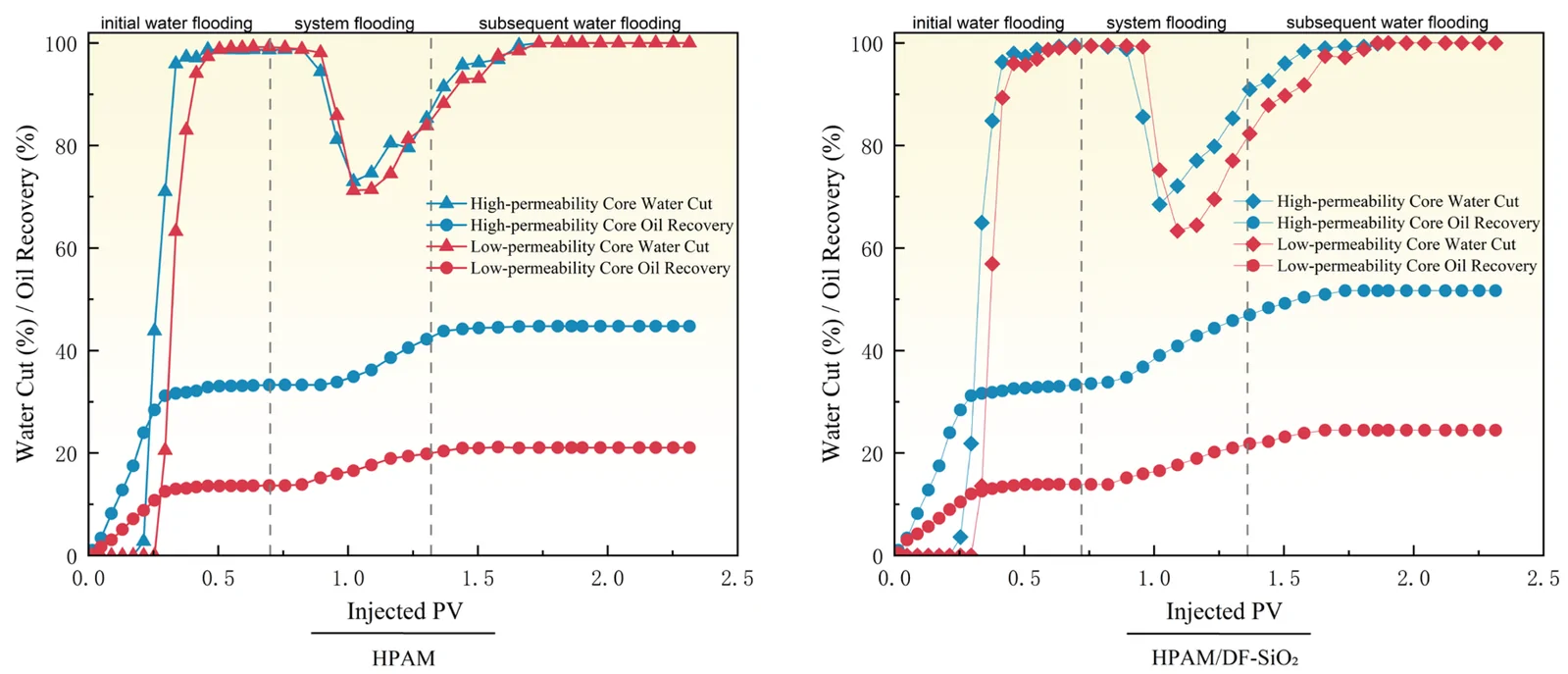
Variation curves of water cut and oil recovery during displacement for different systems.

**Figure 17:**
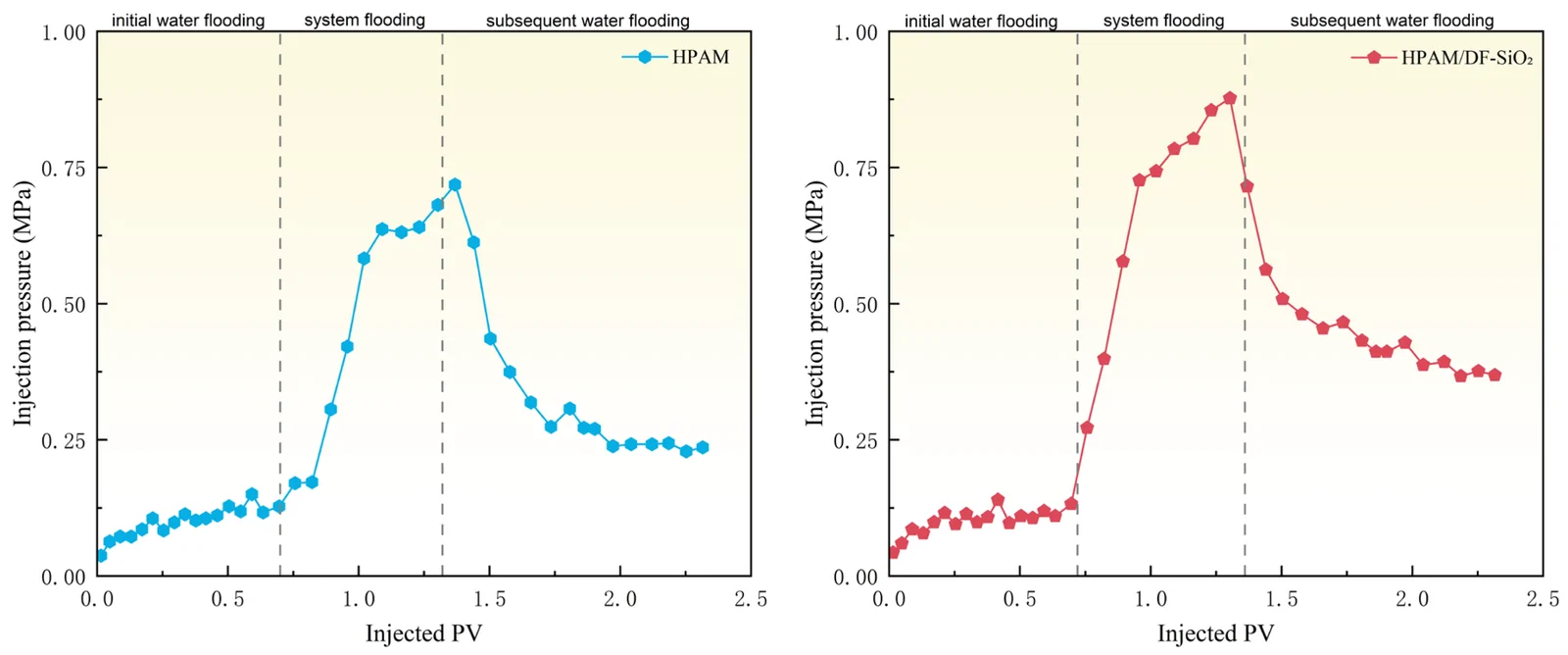
Change curves of displacement pressure in different systems during displacement process.

**Figure 18:**
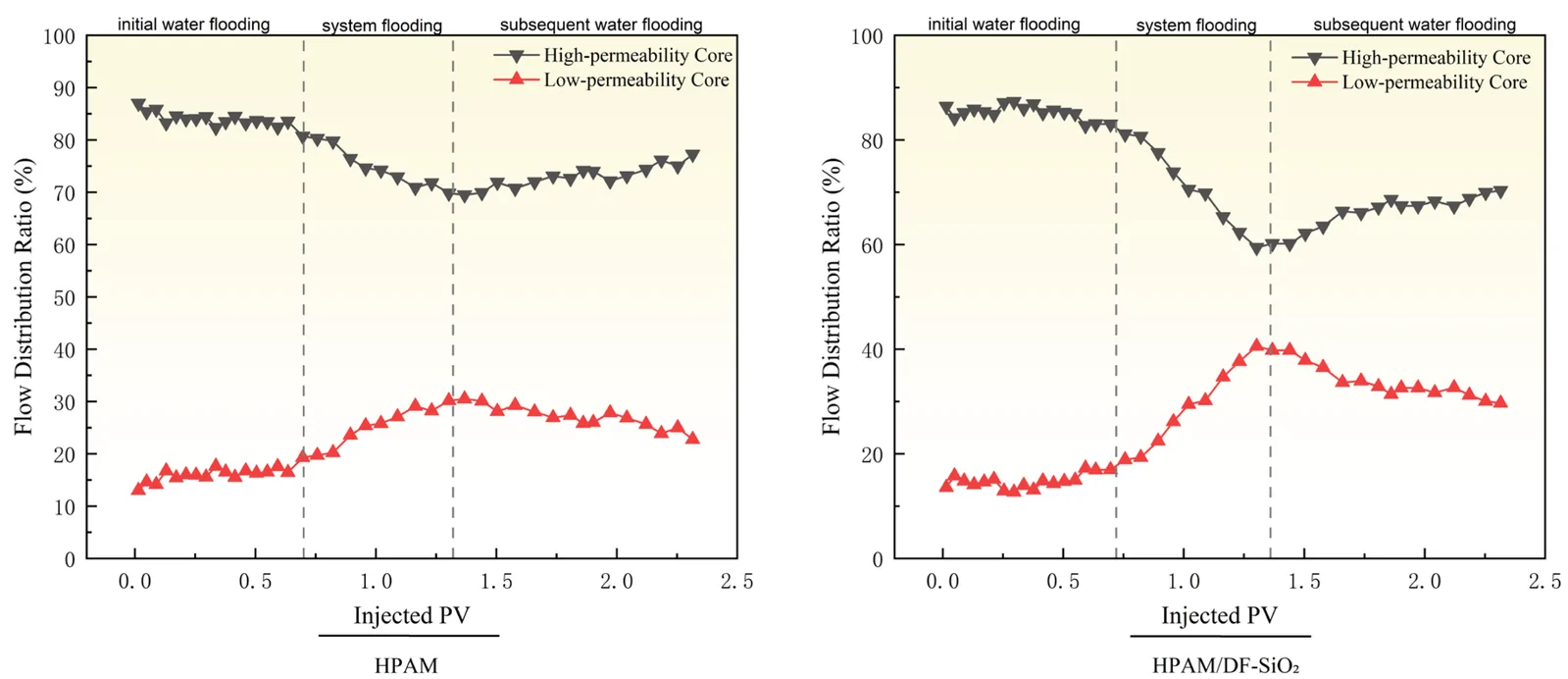
Shunt-rate curves for different systems.

In order to evaluate the effect of HPAM/DF-SiO_2_ two composite gel system on fluid flow distribution in heterogeneous reservoirs, core displacement experiments were carried out in parallel. As shown in Figure 16, Figure 17 and Figure 18, the two systems in the water flooding stage show obvious preferential liquid absorption characteristics of the high permeability layer. The split rate of the high permeability layer of HPAM and HPAM/DF-SiO_2_ system is 83.27% and 84.61%, respectively, and the split rate of the low permeability layer is 16.73% and 15.39%, respectively, indicating that the initial flow distribution is mainly controlled by the permeability difference. This is because the high permeability layer has lower seepage resistance, and it is easier to carry a large proportion of injected fluid under the same injection conditions. With the continuous water flooding, the dominant flow channel is gradually formed, resulting in the fluid entry degree of the low permeability layer is limited. At the same time, the displacement pressure of the two systems is at a low level, and the water content increases rapidly and then tends to be stable, indicating that the fluid mainly migrates along the high permeability channel during the water flooding process.

After entering the composite gel flooding stage, the displacement pressure of the two systems increased significantly (Figure 17), indicating that the injected system raised the flow resistance within the porous medium after entering the core. Previous studies have shown that the flow pressure response of HPAM in medium- and low-permeability cores is closely related to core permeability, polymer properties and injection conditions. The actual flow behavior in porous media is not completely consistent with the bulk rheological response [47,50,51]. In this study, the displacement pressures of the HPAM and HPAM/DF-SiO_2_ systems increased to approximately 0.72 and 0.88 MPa, respectively, and the HPAM/DF-SiO_2_ system exhibited a higher pressure response, indicating that the flow resistance of the composite gel within the porous medium was further enhanced. Corresponding to the pressure variation, obvious inter-layer flow redistribution occurred for both systems. The flow split rate of the high-permeability layer for the HPAM system decreased from 83.27% to 65.77%, while that of the low-permeability layer rose to 34.23%. For the HPAM/DF-SiO_2_ system, the flow split rate of the high-permeability layer was further reduced to 59.43%, and that of the low-permeability layer increased to 40.57% (Figure 18). Meanwhile, the water cut of both systems declined during the polymer flooding stage (Figure 16). Compared with HPAM, the HPAM/DF-SiO_2_ composite gel demonstrated more pronounced suppression of flow in the high-permeability layer and enhanced flow in the low-permeability layer, showing that its high flow resistance can further reduce the flow capacity of the dominant channel and divert more displacing fluid into the low-permeability layer. The action process can be understood as follows: the composite gel preferentially enters the high permeability channel with low flow resistance, and forms a certain retention and network resistance in the pores, which reduces the flow carrying capacity of the original dominant channel, and then promotes the subsequent displacement fluid to redistribute to the low permeability channel. Previous studies have reported that the interaction between the gel network and pores of porous media can produce pore-scale retardation and alter the migration path and local flow characteristics inside pores [52]. By raising seepage resistance and improving mobility control, this type of composite gel can promote the sweep efficiency of low-permeability zones in heterogeneous reservoirs [23,53].

In the subsequent water flooding stage, the displacement pressure of the two systems gradually decreased but did not immediately return to the initial water flooding level (Figure 17), and the flow split rate of the high-permeability layer increased. The flow split rate of the low-permeability layer for the HPAM/DF-SiO_2_ system remained at 29.68%, which was higher than the 25.85% of the HPAM system (Figure 18), indicating that the composite gel still maintained a certain flow control effect within the core. Combined with the core flow recovery results presented in Section 2.3.1, it can be seen that the HPAM/DF-SiO_2_ composite gel can not only generate high flow resistance during displacement, but also retain a certain flow regulation effect in the subsequent water flooding stage, so that the fluid redistribution effect can be maintained to a certain extent.

The stratified production results further verified the flow control effect of the composite gel. The ultimate oil recovery of the high-permeability layer and low-permeability layer for the HPAM/DF-SiO_2_ system reached 51.69% and 25.45%, respectively, which were 6.95 and 4.41 percentage points higher than those of the HPAM system (44.74% and 21.04%). Combined with Figure 16 and Figure 18, it can be seen that the HPAM/DF-SiO_2_ composite gel system not only suppresses the dominant flow in the high-permeability layer, but also raises the flow proportion of the low-permeability layer, allowing more displacing fluid to enter the low-permeability layer and thereby enhancing its displacement efficiency. Previous studies have demonstrated that nano-SiO_2_ and functionalized polymer systems can enhance crude oil recovery by modifying flow characteristics and improving reservoir sweep efficiency [23,27,53].

It can be seen from Figure 16, Figure 17 and Figure 18 that the incorporation of DF-SiO_2_ further evolves the HPAM system from simple viscosity control into a composite gel flow-control system with dynamic network characteristics. The increase in flow resistance of the composite gel within the core corresponds to the reduction in the flow split rate of the high-permeability layer and the rise in the flow split rate of the low-permeability layer, which is further manifested in the simultaneous enhancement of layered oil recovery. A certain pressure and flow split regulation effect is still retained during the subsequent water flooding stage, demonstrating that the flow resistance formed by the network structure possesses a certain persistence. Therefore, the above rheological, structural recovery, and double-tube parallel-core experimental results are mutually consistent, showing that the HPAM/DF-SiO_2_ composite gel can convert its dynamic network structure into flow control capability in porous media. By optimizing fluid distribution between high- and low-permeability layers, the sweep participation of the low-permeability layer is promoted, and the overall displacement performance of the heterogeneous core is improved.

In this study, a permeability ratio of about 5:1 is used, mainly referring to the heterogeneous characteristics of the target block, which can reflect the flow difference between high and low permeability channels and the flow redistribution process after gel injection. For reservoirs with further increased permeability differences, the dominant flow in the high-permeability channel is expected to be more obvious, and the importance of selectively reducing the flow capacity of the dominant channel and promoting subsequent fluid redistribution will also be further enhanced. In the future, further experiments will be carried out by setting a larger permeability ratio to explore the profile control adaptability and flow control effect of the composite gel system under different degrees of heterogeneity.

## 3. Conclusions

(1) Bifunctional modified nano-SiO_2_: KH570@SiO_2_-AMPS/ODMA (DF-SiO_2_) was successfully prepared. The results of FT-IR, TGA, XPS and DLS/Zeta potential showed that the functional structure of AMPS/ODMA was successfully introduced into the surface of SiO_2_, which provided a structural basis for its participation in the construction of HPAM composite gel network.

(2) DF-SiO_2_ significantly improved the viscoelasticity and stability of HPAM. Under the condition of 90 °C and 20,000 mg/L salinity, the viscosity of HPAM/DF-SiO_2_ composite gel system reached 29.7 mPa·s. After 90 days of aging, the viscosity remained at 23.0 mPa·s, and the viscosity recovered to 28.1 mPa·s after 72 h of standing following shearing, indicating that the HPAM/DF-SiO_2_ composite gel system had good temperature and salt tolerance and structural recovery ability.

(3) Core flow and double-tube parallel displacement experiments show that the HPAM/DF-SiO_2_ composite gel system has better flow control and enhanced oil recovery. After shearing and standing for 5 days, RF recovered to 10.8; after polymer flooding, the diversion rates of high and low permeability layers are 59.43% and 40.57% respectively, and the final recovery rates of high and low permeability layers are 51.69% and 25.45% respectively, which are 6.95 and 4.41 percentage points higher than that of HPAM.

(4) Although the HPAM/DF-SiO_2_ composite gel system exhibits good temperature and salt tolerance and heterogeneous reservoir regulation ability, the current research mainly focuses on the indoor experimental scale, and its long-distance migration and continuous regulation ability under complex reservoir conditions and economic evaluation still need further study.

## 4. Materials and Methods

### 4.1. Experimental Materials

Nano-SiO_2_ (particle size: 30–40 nm, specific surface area: 350 m^2^/g, purity: 99.5%), γ-methacryloxypropyltrimethoxysilane (KH570, analytically pure), 2-acrylamido-2-methylpropanesulfonic acid (AMPS, analytically pure) and octadecyl methacrylate (ODMA, analytically pure, containing stabilizer MEHQ, Aladdin; liquefied by heating in a 30 °C water-bath before use) were the main raw materials for preparing bifunctional nano-components. Partially hydrolyzed polyacrylamide (HPAM, anionic, degree of hydrolysis 25%, molecular weight 20 × 10^6^, solid content 89.2 wt%) was adopted as the polymer matrix. Ammonium persulfate (APS, analytically pure) served as the free-radical initiator for the in situ grafting of functional monomers onto the nano-SiO_2_ surface. Anhydrous ethanol (analytically pure, 99.5%), 1,4-dioxane (analytically pure) and acetic acid (analytically pure) were applied in material preparation. Potassium bromide (KBr, spectrally pure) was used for Fourier-transform infrared spectroscopy test. Calcium chloride dihydrate (CaCl_2_·2H_2_O), magnesium chloride hexahydrate (MgCl_2_·6H_2_O) and sodium chloride (NaCl) were used to prepare simulated formation water. High-purity nitrogen (N_2_, purity ≥ 99.999%) provided inert-atmosphere protection for the grafting reaction.

The experimental water was simulated formation water with a target salinity of 20,000 mg/L. The main-ion compositions were Ca^2+^: 420 mg/L, Mg^2+^: 230 mg/L, Na^+^: 6450 mg/L and Cl^−^: 12,750 mg/L. The simulated formation water was prepared according to the target ion concentrations and diluted to 1 L using deionized water as solvent.

The experiment used degassed crude oil from the target reservoir. The dynamic viscosity of the crude oil was 6.3 mPa·s at 90 °C (density: 0.832 g/cm^3^ at 20 °C; API gravity: 38.1°; SARA composition: saturates 64.08 wt%, aromatics 17.25 wt%, resins 16.32 wt%, and asphaltenes 2.35 wt%).

Natural Berea sandstone was adopted as core samples. The core dimensions were 30 cm × 4.5 cm × 4.5 cm, with permeabilities of approximately 10 × 10^−3^ μm^2^ and 50 × 10^−3^ μm^2^, respectively. They were used to evaluate the flow-control and displacement performance of the composite system in porous media with different permeabilities.

### 4.2. Experiment Instrument

The main experimental instruments included DF-101S thermostatic magnetic stirrer (Shanghai Lichen Bonxi Instrument Technology Co., Ltd., Shanghai, China), KQ-300DE numerical-control ultrasonic cleaner (Kunshan Ultrasonic Instrument Co., Ltd., Kunshan, China), TD5 desktop low-speed centrifuge (Shanghai Luxiangyi Centrifuge Instrument Co., Ltd., Shanghai, China), DZF-6020 vacuum-drying oven (Shanghai Boxun Industrial Co., Ltd., Shanghai, China), FA2204 electronic analytical balance (Shanghai Lichen Bonxi Instrument Technology Co., Ltd., Shanghai, China), RW20 overhead mechanical stirrer (IKA, Staufen, Germany), WB3000 high-speed shear disperser (WIGGENS, Wuppertal, Germany), and UV-1800 ultraviolet–visible spectrophotometer (Shimadzu, Kyoto, Japan). These instruments were mainly applied for sample weighing, dispersion, stirring, centrifugal washing, drying, shear treatment and concentration measurement.

The structure and properties of the samples were characterized by Nicolet 6700 Fourier-transform infrared spectrometer (Thermo Fisher Scientific, Waltham, MA, USA), Q50 thermogravimetric analyzer (TA Instruments, New Castle, DE, USA), ESCALAB 250 Xi X-ray photoelectron spectrometer (Thermo Fisher Scientific, Waltham, MA, USA), Zetasizer Nano ZS90 nanoparticle-size and Zeta potential analyzer (Malvern Panalytical, Malvern, UK), Discovery HR-1 rotational rheometer (TA Instruments, New Castle, DE, USA), Brookfield DV-III Ultra rotational viscometer (Brookfield, Middleboro, MA, USA), and Quanta FEG450 field-emission scanning electron microscope (FEI, Hillsboro, OR, USA).

Core-flooding experiments were performed using an ISCO constant-flow pump (Teledyne ISCO, Lincoln, NE, USA) and a laboratory core-displacement experimental system (including thermostat, core holder, intermediate container, pressure sensor, gas–liquid separator, graduated cylinder, etc., Jiang Suyou Scientific Instrument Co., Ltd., Haian, China). This setup was used to conduct experiments on flow-recovery behavior of the composite system in porous media and heterogeneous core displacement tests.

### 4.3. Preparation of Bifunctional Modified Nano-SiO_2_ and Construction of HPAM/DF-SiO_2_ Composite Gel System

#### 4.3.1. Preparation of KH570@SiO_2_-AMPS/ODMA

**Figure 19:**
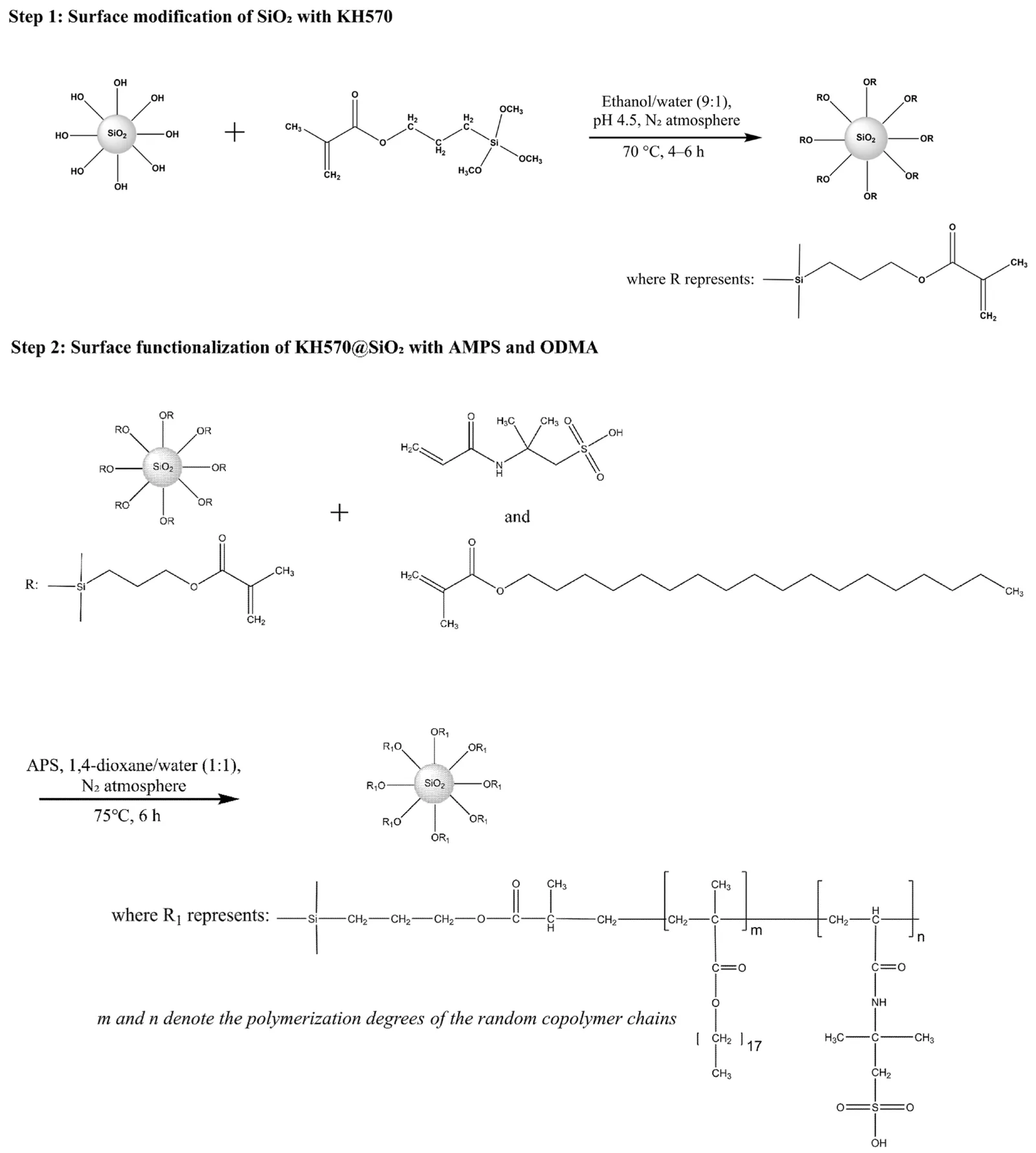
Preparation route of KH570@SiO_2_-AMPS/ODMA.

(1) As illustrated in Figure 19, the surface of nano-SiO_2_ is rich in silanol groups (Si-OH), which can react with silane coupling agents [25]. To introduce further-reactive interfacial bonding sites onto the SiO_2_ surface, KH570 was employed for surface modification of nano-SiO_2_.

The detailed preparation procedure is described below. First, 1.00 g of nano-SiO_2_ (specific surface area: 350 m^2^/g) was weighed and added into 100 mL anhydrous-ethanol/deionized-water mixed solution (volume ratio 9:1). The mixture was ultrasonically dispersed at room temperature for 30 min with an ultrasonic power of 120 W to obtain a homogeneous SiO_2_ suspension. Afterwards, acetic acid was added drop-wise to adjust the system pH to 4.5, so as to promote hydrolysis of the methoxy groups in KH570. Then 0.10 g KH570 was added, corresponding to a SiO_2_-to-KH570 mass ratio of 10:1. High-purity N_2_ was purged into the reaction system for 30 min. Under N_2_ atmosphere protection, the mixture was stirred at 300 rpm for 4 h in a thermostatted water-bath at 70 °C.

After the reaction finished, the system was cooled down to room temperature and centrifuged at 8000 rpm for 10 min, and the supernatant was decanted. The collected precipitate was redispersed in anhydrous ethanol and centrifuged repeatedly. This washing operation was performed three times to remove residual unreacted KH570. After washing, the product was transferred to a vacuum-drying oven, vacuum-dried at 60 °C for 24 h, and then ground into fine powder to obtain KH570-modified nano-SiO_2_ (KH570@SiO_2_).

The methoxy groups in KH570 molecules undergo hydrolysis to generate silanol groups, which further undergo dehydration–condensation with surface hydroxyl groups on SiO_2_ particles, forming Si-O-Si linkages on the particle surface. Meanwhile, the carbon-carbon double bond in the terminal methacryloyl group remains intact, providing reactive sites for subsequent free-radical polymerization of functional monomers [20,25].

(2) Preparation of KH570@SiO_2_-AMPS/ODMA. Functional monomers AMPS and ODMA were simultaneously grafted onto KH570-modified nano-SiO_2_ via an in situ free-radical grafting method [17,54].

First, 1.00 g of KH570@SiO_2_ was added into 80 mL 1,4-dioxane/deionized-water mixed solvent (volume ratio 1:1), followed by ultrasonic dispersion at room temperature for 20 min to form a homogeneous suspension. ODMA was liquefied by heating in a 30 °C water-bath prior to use. Afterwards, 0.96 g AMPS and 0.24 g ODMA were introduced into the system. The total monomer mass was 1.20 g, with an AMPS-to-ODMA mass ratio of 4:1 and a total-monomer-to-KH570@SiO_2_ mass ratio of 1.2:1. The monomers were fully dispersed under continuous magnetic stirring.

In this study, the effects of different AMPS/ODMA mass ratios on the apparent viscosity of the composite gel system were investigated in the previous formulation screening. The initial setting of AMPS/ODMA mass ratio refers to the existing research on the construction of associative polymer systems by AMPS and long-chain hydrophobic monomers [55], and is determined based on the previous synthesis experience of this study. In the case of consistent total monomer dosage and other synthesis conditions, the AMPS/ODMA mass ratio was set to 1:1, 2:1, 3:1, 4:1, 5:1 and 6:1, and pre-screening was performed under target reservoir conditions. The results showed that the apparent viscosity of the 3:1 system was the highest, which was 30.4 mPa·s, while that of the 4:1 system was 29.7 mPa·s, and the difference between the two was only about 2.3%. Considering that the apparent viscosity difference between the two ratios is small, and the 4:1 system has a higher relative ratio of AMPS, which is conducive to further exerting the salt tolerance of AMPS under high-salinity conditions. Therefore, 4:1 is selected as a representative ratio for subsequent experiments.

Subsequently, high-purity N_2_ was purged into the reaction system for 30 min, and the system was heated to 75 °C. A total of 0.0144 g APS was dissolved in 2 mL deionized water and rapidly injected into the reaction mixture. The dosage of APS accounted for 1.2 wt% of the total monomer mass. The mixture was stirred at 75 °C for 6 h under N_2_ atmosphere protection. Thermal decomposition of APS generates free radicals, which initiate the free-radical grafting reaction between functional monomers and surface-active double bonds, and an organic functional layer containing AMPS- and ODMA-derived moieties is constructed on the surface of nano-SiO_2_.

Upon completion of the reaction, the system was cooled to room temperature and slowly poured into anhydrous ethanol (10-fold volume of the reaction solution) for precipitation. Afterwards, centrifugation was performed at 8000 rpm for 10 min, and the supernatant was decanted. The collected precipitate was redispersed and washed with anhydrous-ethanol/deionized-water mixed solvent (volume ratio 1:1). The washing procedure was repeated three times to eliminate residual monomers and soluble homopolymers. The purified product was vacuum-dried at 60 °C for 24 h and ground to yield bifunctionally modified nano-SiO_2_ (KH570@SiO_2_-AMPS/ODMA, denoted as DF-SiO_2_), which was sealed for storage.

Among them, ODMA supplies long-chain octadecyl groups and acts as hydrophobic-association sites [17,18,19]. The sulfonate groups introduced by AMPS impart high hydrophilicity and ion tolerance to the particle surface, improving the stability of composite particles under high-salinity conditions [18,23,24]. By incorporating these two kinds of functional structures onto KH570@SiO_2_ surface, DF-SiO_2_ possesses both dynamic-association capability and environmental-stability regulation performance, and provides an interfacial foundation for its participation in constructing the HPAM polymer network.

#### 4.3.2. Preparation of HPAM and Various Composite-Gel Systems

The HPAM concentration for all composite systems in this work was fixed at 1500 mg/L. The HPAM dry powder used in the experiment is an industrial polymer specially developed for oil-field displacement, supplied by PetroChina Daqing Refining and Chemical Company (Kunlun Brand, Daqing, China). It has a molecular weight of 20 × 10^6^ g/mol, a degree of hydrolysis of 25 mol%, and a solid content of 89.2 wt%.

HPAM powder was slowly sprinkled onto the surface of deionized water under stirring at 300 rpm to prevent pronounced vortices that would cause polymer agglomeration. The HPAM stock solution at 1500 mg/L was prepared by continuous stirring at room temperature for 6 h until full dissolution, followed by static maturation for 24 h.

Nano-SiO_2_, KH570@SiO_2_ and DF-SiO_2_ were dispersed in deionized water to prepare nanoparticle suspensions with a mass concentration of 10 g/L. Ultrasonic treatment was applied for 30 min to yield homogeneous dispersions.

Under gentle stirring at 500 rpm, the above nanoparticle suspension was slowly added into the HPAM stock solution. The nanoparticle dosage was fixed at 0.5 wt% (based on the dry mass of HPAM). For 1 L of 1500 mg/L HPAM stock solution, 0.75 mL of the 10 g/L nanoparticle suspension was introduced to achieve the target nanoparticle loading of 0.5 wt%. After completion of addition, stirring was maintained for another 2 h to achieve thorough dispersion of nanoparticles within the polymer matrix.

Four stock solutions were prepared: pure HPAM, HPAM/SiO_2_, HPAM/KH570@SiO_2_ and HPAM/KH570@SiO_2_-AMPS/ODMA (HPAM/DF-SiO_2_). The HPAM mass concentration was kept constant at 1500 mg/L for all composite systems. All freshly prepared samples were sealed and allowed to stand for 24 h. Subsequent rheological and stability measurements were performed after full equilibrium of each system.

Within these systems, HPAM serves as the polymer-network matrix, while nano-SiO_2_ and its functionalized derivatives act as interfacial components that participate in spatial interactions among polymer chains. For the HPAM/DF-SiO_2_ system, surface-bound AMPS and ODMA moieties provide environmental stability regulation and hydrophobic-association sites, respectively. They facilitate the formation of dynamic physical cross-links between polymer chains, and consequently build a composite network structure with nano-interface nodes.

### 4.4. Characterization and Performance Evaluation of HPAM/DF-SiO_2_ Composite Gel System

#### 4.4.1. FT-IR Characterization

The chemical structures of differently modified nano-SiO_2_ samples were characterized using a Nicolet 6700 Fourier-transform infrared spectrometer (Thermo Fisher Scientific, Waltham, MA, USA). Prior to measurement, the samples were thoroughly dried, mixed with KBr at a mass ratio of approximately 1:100, ground finely and pressed into transparent pellets. Scanning was performed over the wavenumber range of 4000–400 cm^−1^ with a spectral resolution of 4 cm^−1^, and 32 cumulative scans were acquired. The infrared absorption spectra were recorded to analyze the introduction of surface functional groups and functional segments on SiO_2_.

#### 4.4.2. TGA Characterization

Thermal-stability measurements of the samples were carried out on a Q50 thermogravimetric analyzer (TA Instruments, New Castle, DE, USA). Dried samples of 5–10 mg were weighed into alumina crucibles and measured under a high-purity nitrogen atmosphere. The temperature was raised from room temperature to 800 °C at a heating rate of 10 °C·min^−1^. The mass-versus-temperature curves of the samples were recorded.

#### 4.4.3. XPS Characterization

The surface elemental composition and chemical states of samples were characterized with an ESCALAB 250 Xi X-ray photoelectron spectrometer (Thermo Fisher Scientific, Waltham, MA, USA). A monochromatic Al Kα X-ray source with an excitation power of 150 W was employed. The base vacuum of the analysis chamber was better than 1 × 10^−9^ mbar. Binding energies were calibrated using the C 1s peak of adventitious carbon (284.8 eV). The survey-scan range was 0–1200 eV with a step size of 1 eV. By analyzing the positions and relative intensities of characteristic elemental peaks, the surface elemental compositions and their variations at different modification stages were compared. This provides surface-chemical evidence for the successful grafting of AMPS and ODMA functional groups onto nano-SiO_2_.

#### 4.4.4. DLS and Zeta Potential

The hydrodynamic particle size and Zeta potential of various modified nano-SiO_2_ dispersions were measured using a Zetasizer Nano ZS90 nanoparticle-size and Zeta potential analyzer. Prior to testing, samples were dispersed in deionized water under ultrasonication for 10 min, and measurements were performed at 25 ± 0.5 °C. DLS was adopted to characterize the hydrodynamic diameter of particles in aqueous medium, which reflects particle-size variations induced by the grafted organic surface layer. Zeta potential characterizes the surface-charge properties of particles, indirectly indicating electrostatic repulsion and dispersion tendency among particles, and evaluating the anti-agglomeration dispersion performance in aqueous solution [26]. Each sample group was tested in triplicate, and the average value was taken as the final result.

#### 4.4.5. System Formulation Screening

The influence of DF-SiO_2_ dosage (0.1–1.0 wt%, based on the dry mass of HPAM) on the apparent viscosity of composite systems was investigated at 90 °C under a salinity of 20,000 mg/L, so as to determine the working nanoparticle loading for subsequent experiments. Viscosity measurements were carried out with a Brookfield DV-III Ultra rotational viscometer equipped with a UL rotor at a shear rate of 7.34 s^−1^. Each formulation was measured in triplicate, with the mean value taken as the result.

#### 4.4.6. Evaluation of System Dispersion Stability

The static-sedimentation method was used to evaluate the short-term dispersion stability of the HPAM/DF-SiO_2_ composite system. Composite systems with different DF-SiO_2_ dosages were prepared, sealed, and kept static at a constant temperature of 90 °C for 96 h. Supernatant aliquots were collected at a fixed position below the liquid surface at 0, 12, 24, 48, 72 and 96 h. The absorbance was measured at the characteristic wavelength by an ultraviolet–visible spectrophotometer. The concentration of dispersed DF-SiO_2_ was calculated according to the pre-established concentration–absorbance standard curve. With the initial concentration C_0_ as the reference, the relative concentration C/C_0_ was computed to characterize the dispersion-stability degree of nanoparticles in the system. Each sample group was measured in triplicate, with results presented as mean ± standard deviation.

#### 4.4.7. Rheological Property Measurement

The viscoelastic responses of various HPAM composite systems were measured using a Discovery HR-1 rotational rheometer (TA Instruments, New Castle, DE, USA). The tested systems included HPAM, HPAM/SiO_2_, HPAM/KH570@SiO_2_, and HPAM/DF-SiO_2_. Prior to measurement, samples were thermally equilibrated at 90 °C for 10 min.

First, strain-sweep tests were conducted to identify the linear viscoelastic region (LVR) for each system. Strain-sweep measurements were performed at 90 °C with an angular frequency of 10 rad·s^−1^. The results indicated that 1% strain fell within the LVR for all systems; therefore, 1% strain was adopted as the fixed strain for the subsequent oscillatory frequency-sweep tests. A 50 mm diameter parallel-plate geometry was employed, with the test gap set to 1 mm. The angular frequency ranged from 0.1 to 100 rad·s^−1^. The storage modulus (G′) and loss modulus (G″) were recorded as functions of frequency to evaluate the viscoelastic behavior and features of the internal dynamic-network structure of composite systems. For quantitative comparison among different systems, the reported G′ and G″ values in this work were extracted from test data obtained at 100 rad·s^−1^.

#### 4.4.8. Temperature-Resistance and Salt-Resistance Performance Test

Viscosity measurements were performed using a Brookfield DV-III Ultra rotational viscometer (Brookfield, USA), following the industry standard SY/T 5862-2020 [56]. A UL rotor was adopted with a fixed shear rate of 7.34 s^−1^.

For the temperature-resistance test, various system solutions with a mass concentration of 1500 mg/L were prepared. Under a salinity of 20,000 mg/L, the apparent viscosity was measured at 30, 50, 70, 80, 90, 100 and 110 °C.

For the salt-resistance test, the test temperature was fixed at 90 °C. Experimental systems were prepared with simulated formation water of different salinities (10,000, 15,000, 20,000, 25,000 and 30,000 mg/L), and the variations in apparent viscosity under different conditions were determined.

Each condition for both types of experiments was tested in triplicate, and the mean value was adopted as the result.

#### 4.4.9. Post-Shear Structural-Recovery Performance Test

A Brookfield DV-III Ultra rotational viscometer was used to evaluate the structural retention and recovery behavior of different systems after intense shearing. Solutions of various systems at 1500 mg/L were prepared, and their initial apparent viscosity was measured at 90 °C. Afterwards, the sample was transferred to a WB3000 high-speed shear disperser and continuously sheared at 5000 r·min^−1^ for 10 min. Immediately after shearing, the post-shear apparent viscosity was measured at 90 °C. The sample was then kept static at 90 °C for recovery, and the apparent viscosity was recorded at 24, 48 and 72 h to assess the structural-recovery capacity after single shear disturbance.

To further investigate the repeatability of recovery behavior under continuous shearing, three successive shear-recovery cycle tests were carried out. Identical shear conditions (5000 r·min^−1^, 10 min) were applied in each cycle. The apparent viscosity was measured right after shearing, followed by static recovery at 90 °C for 24 h and viscosity measurement. This procedure was repeated for three cycles. The viscosity reduction and recovery degree in each cycle were compared. Each sample was tested in triplicate, and the mean was taken as the result.

#### 4.4.10. Long-Term Stability Test

Solutions of different systems were sealed under oxygen-free conditions and aged at 90 °C in the dark for 90 d. During aging, aliquots were taken at 1, 7, 15, 30, 60 and 90 d for apparent-viscosity measurement, and visual changes in the systems were recorded. The viscosity retention ratio was calculated by comparing the viscosity at 90 d with the initial viscosity. Three parallel aging experiments were conducted for each system (*n* = 3), and the results were averaged.

#### 4.4.11. FESEM

A Quanta FEG450 field-emission scanning electron microscope was employed to observe the micro-morphology of samples. Prior to observation, samples were freeze-dried and gold-sputtered to improve electrical conductivity. FESEM micrographs were acquired at an accelerating voltage of 30 kV to characterize micro-network features and structural continuity of different systems. Representative micrographs were selected for comparative analysis.

#### 4.4.12. Core Flow-Recovery Experiment

Natural Berea sandstone cores were used for core flow-recovery experiments. All cores had uniform dimensions of length × width × height = 30 cm × 4.5 cm × 4.5 cm, with permeability maintained at 50 × 10^−3^ μm^2^. The experimental temperature was 90 °C, the salinity of the test water was 20,000 mg/L, and the injection flow rate was set to 0.5 mL/min.

The HPAM and HPAM/DF-SiO_2_ systems were tested under three sample states: original, post-sheared, and recovered. The shear-treatment procedure was identical to that described in Section 4.4.9. For recovered-state samples, after shearing, the samples were statically held at 90 °C for 5 d prior to core-flow tests, so as to evaluate the seepage-structure recovery capability of the system after shear disturbance.

During the experiment, 0.5 PV of polymer system was first injected into the core, followed by continuous injection of simulated formation water. The pressure was considered stable when the pressure differential across the core gradually leveled off and fluctuated within a narrow range. Variations in injection pressure were recorded throughout the test. The resistance factor (*RF*) and residual resistance factor (*RRF*) were calculated according to Equations (1) and (2):

(1) $$\mathrm{RF}=\frac{\Delta P_{p}}{\Delta P_{w}}$$

(2) $$\mathrm{RRF}=\frac{\Delta P_{\mathrm{aw}}}{\Delta P_{w}}$$

where Δ*P_w_*, Δ*P_p_* and Δ*P_aw_* are the stable pressure differentials for water-flooding, polymer-flooding and subsequent water-flooding stages, respectively. These parameters were used to evaluate the core-flow characteristics of the systems under different states. Three parallel experiments were performed for each system (*n* = 3), and the results were reported as the mean values with standard deviations.

#### 4.4.13. Indoor Double-Tube Parallel-Core Displacement Experiment

**Figure 20:**
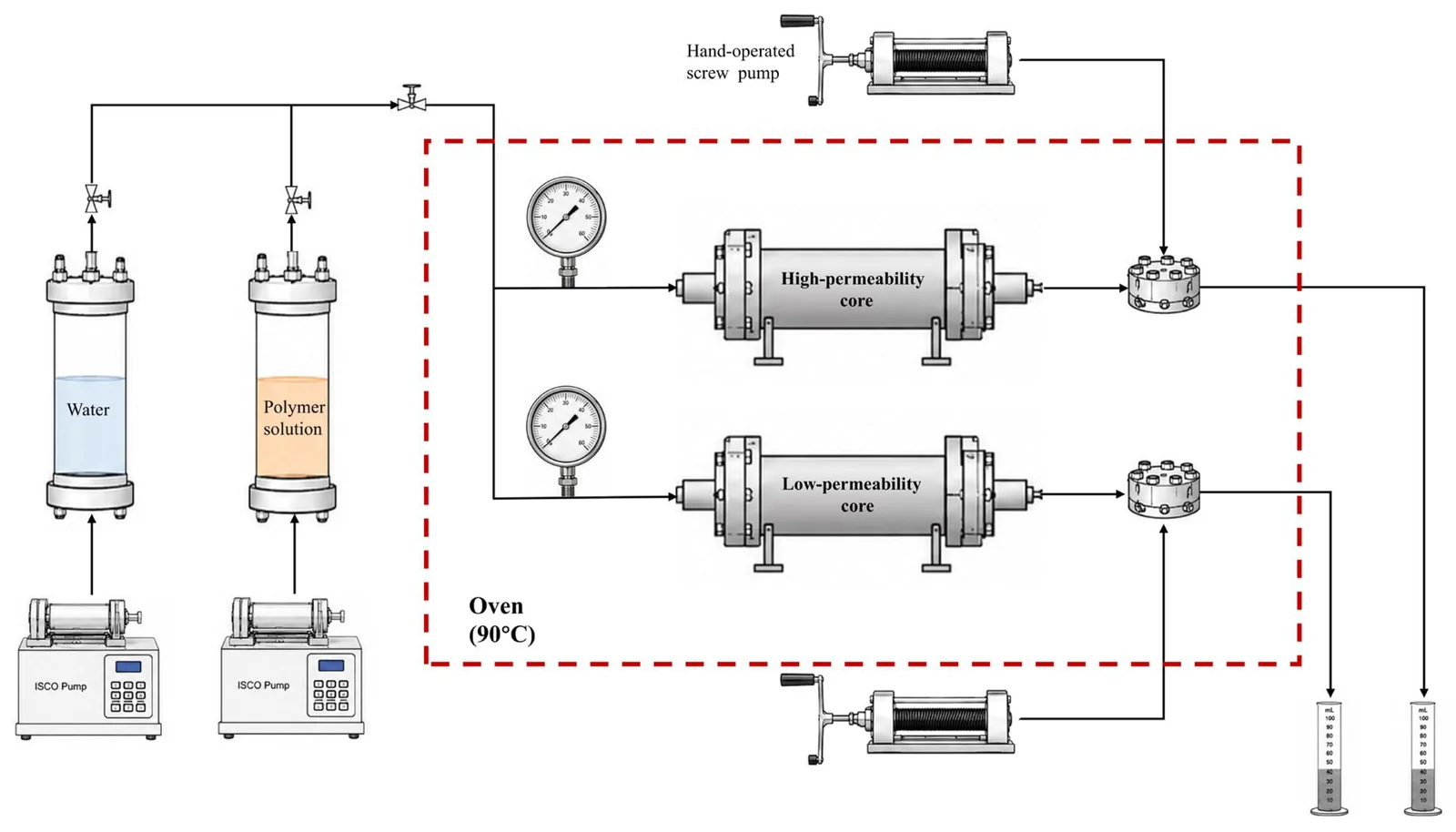
Schematic diagram of double-tube parallel-core displacement experiment.

In order to evaluate the fluid flow regulation and enhanced oil recovery capacity of different gel systems, heterogeneous reservoir displacement experiments were carried out using a double-tube parallel-core displacement device. Parallel high-permeability and low-permeability cores were adopted, with permeabilities of approximately 50 × 10^−3^ μm^2^ and 10 × 10^−3^ μm^2^, respectively. The experimental device was placed in a constant-temperature environment of 90 °C, and an ISCO constant-flow pump was used for fluid injection at a fixed rate of 0.5 mL/min, as shown in Figure 20. Before the experiment, the double-tube cores were vacuumized and saturated with simulated formation water, and the pore volume and water permeability of the cores were measured. Subsequently, the cores were saturated with simulated crude oil and aged at 90 °C for 24 h. The initial oil saturation was calculated as the ratio of the saturated crude oil volume to the core pore volume:

(3) $$S_{\mathrm{oi}}=\frac{V_{o}}{V_{p}}\times 100\%$$

where *S*_oi_ initial oil saturation, %; V_o_ volume of saturated crude oil, mL; V_p_ core pore volume, mL.

The displacement experiment includes three stages: water flooding, polymer flooding and subsequent water flooding. Firstly, water flooding is carried out until the composite water cut reaches 98%. Subsequently, 0.6 PV of HPAM or HPAM/DF-SiO_2_ composite system was injected respectively. After chemical flooding, simulated formation water was continuously injected until the composite water cut reached 98% again. During the experiment, the injection pressure, liquid production, oil production and water cut of high- and low-permeability cores were recorded in real time, and the separate recovery factor for high- and low-permeability layers as well as the overall recovery factor were calculated respectively. The recovery factor calculation formula is:

(4) $$E_{R}=\frac{\sum V_{\mathrm{op}}}{V_{\mathrm{oi}}}\times 100\%$$

In the formula E_R_ is the recovery factor, %; $\sum V_{\mathrm{op}}$ is the cumulative oil production, mL; V_oi_ is the volume of saturated crude oil in the core, mL.

Limitations of this study: This work is a laboratory core-scale displacement evaluation. Limited-sized natural Berea sandstone cores were used, and the experiments were conducted under ideally controlled conditions with constant temperature and constant flow rate. There are certain differences compared with the complex conditions of actual reservoirs, such as strong heterogeneity, formation stress, multiphase interfacial effects and long-term fluid transport. The experimental results are mainly used to qualitatively and semi-quantitatively reveal the fluid flow regulation mechanism and enhanced oil recovery mechanism of the composite gel system. Three parallel displacement experiments were performed for each system.

## Figures and Tables

**Table 1:** Power-law fitting results of 1–10 rad·s^−1^ frequency scanning data at 90 °C and 1% strain.

System	*n* ^′^	*R*^2^ (for *G*′)	*n*″	*R*^2^ (for *G*″)
HPAM	0.158	0.9975	0.152	0.9821
HPAM/SiO_2_	0.261	0.9981	0.268	0.9968
HPAM/KH570@SiO_2_	0.315	0.9820	0.232	0.9881
HPAM/DF-SiO_2_	0.269	0.9752	0.140	0.9878

## Data Availability

The original contributions presented in this study are included in the article. Further inquiries can be directed to the corresponding author.
